# A Phylogenetically Informed Comparison of GH1 Hydrolases between Arabidopsis and Rice Response to Stressors

**DOI:** 10.3389/fpls.2017.00350

**Published:** 2017-03-24

**Authors:** Yun-Ying Cao, Jing-Fang Yang, Tie-Yuan Liu, Zhen-Feng Su, Fu-Yuan Zhu, Mo-Xian Chen, Tao Fan, Neng-Hui Ye, Zhen Feng, Ling-Juan Wang, Ge-Fei Hao, Jianhua Zhang, Ying-Gao Liu

**Affiliations:** ^1^State Key Laboratory of Crop Biology, College of Life Science, Shandong Agricultural UniversityTaian, China; ^2^College of Life Sciences, Nantong UniversityNantong, China; ^3^Ministry of Agricultural Scientific Observing and Experimental Station of Maize in Plain Area of Southern Region, Nantong UniversityNantong, China; ^4^College of Chemistry, Central China Normal UniversityWuhan, China; ^5^School of Life Sciences and State Key Laboratory of Agrobiotechnology, The Chinese University of Hong KongShatin, Hong Kong; ^6^Shenzhen Research Institute, The Chinese University of Hong KongShenzhen, China; ^7^Jiangsu Entry-exit Inspection And Quarantine BureauNanjing, China

**Keywords:** β-glucosidase, gene expression, multiple alignment, promoter, phylogenetic analysis, stress treatments

## Abstract

Glycoside hydrolases Family 1 (GH1) comprises enzymes that can hydrolyze β-O-glycosidic bond from a carbohydrate moiety. The plant GH1 hydrolases participate in a number of developmental processes and stress responses, including cell wall modification, plant hormone activation or deactivation and herbivore resistance. A large number of members has been observed in this family, suggesting their potential redundant functions in various biological processes. In this study, we have used 304 sequences of plant GH1 hydrolases to study the evolution of this gene family in plant lineage. Gene duplication was found to be a common phenomenon in this gene family. Although many members of GH1 hydrolases showed a high degree of similarity in Arabidopsis and rice, they showed substantial tissue specificity and differential responses to various stress treatments. This differential regulation implies each enzyme may play a distinct role in plants. Furthermore, some of salt-responsive Arabidopsis GH1 hydrolases were selected to test their genetic involvement in salt responses. The knockout mutants of *AtBGLU1* and *AtBGLU19* were observed to be less-sensitive during NaCl treatment in comparison to the wild type seedlings, indicating their participation in salt stress response. In summary, Arabidopsis and rice GH1 glycoside hydrolases showed distinct features in their evolutionary path, transcriptional regulation and genetic functions.

## Introduction

As sessile organisms, plants encounter a variety of abiotic stresses during their life cycle. For example, cold or freezing stresses are major environmental factors that limit the distribution and productivity of plant species (Du et al., 2010). Low temperature may result in a series of detrimental phenomenon to plants such as pollen sterility and low membrane fluidity. Drought stress leads to water depletion of plant cells, which will destroy the bilayer of plant cell membrane and thereby increase the membrane permeability (Du et al., 2013). High salinity severely hinders crop yield. Its secondary effects, such as osmotic imbalance and ion toxicity, are adverse to plant development (Munns and Tester, 2008). In addition, hyper-osmolarity represents a generalized secondary stress response resulting from cold, drought and high salinity (Yamaguchi-Shinozaki and Shinozaki, 2005).

Glycoside hydrolases or glycosidase are widely distributed enzymes that can hydrolyze the glycosidic bond of carbohydrates (Chandrasekar et al., 2014). To date, 135 families of these enzymes have been classified according to the CAZy database (Xu et al., 2004; Cantarel et al., 2009; Lombard et al., 2014). Animal glycosidases have been shown to play tremendous roles in human disease such as Diabetes and Gaucher disease etc. (Körschen et al., 2013; Antu et al., 2014), indicating their crucial functions in human health. In comparison to animal glucosidases, higher plants contain a large number of genes encoding glycosidases (Xu et al., 2004; Opassiri et al., 2006), suggesting their extensive functional redundancy or differential responsiveness in plants.

In the last decades, although GH1 hydrolases from model plants Arabidopsis, rice and maize have been extensively studied (Xu et al., 2004; Opassiri et al., 2006; Gomez-Anduro et al., 2011; Zhao et al., 2012), their evolutionary origin from plant lineage remains unclear. Furthermore, the substrate specificities of many GH1 hydrolases have been tested (Wang et al., 2011; Luang et al., 2013; Rouyi et al., 2014) and the crystal structures of several representative members have been solved to further validate the molecular mechanisms of their enzymatic cleavage (Seshadri et al., 2009; Roston et al., 2014), which greatly promote the research in this field. However, relatively few functional studies in plants using genetic approach have been reported. Several plant GH1 hydrolases have been reported to play important roles in diverse biological processes including defense against herbivore, cell wall lignification, phytohormone activation and secondary metabolism (Wang et al., 2011; Chapelle et al., 2012; Xu et al., 2012; Zhou et al., 2012; Miyahara et al., 2013). For instance, AtBGLU42 has been reported to modulate iron deficiency response and regulate rhizobacteria-induced systemic resistance in Arabidopsis (Zamioudis et al., 2014). Four members AtBGLU34, AtBGLU35, AtBGLU37, and AtBGLU38, also known as β-S-glucosidases (myrosinases) have been demonstrated to hydrolyze glucosinolate and participate in plant defense responses (Barth and Jander, 2006; Wittstock and Burow, 2010). In addition, two BGLUs, AtBGLU18, and AtBGLU33 have been reported to hydrolyze the glucose ester of abscisic acid (ABA-GE), a storage form of plant hormone ABA (Lee et al., 2006; Xu et al., 2012). Thus, they play important regulatory role in ABA responses. Maize ZmBGLU1 has been characterized to release active cytokinins by hydrolyzing cytokinin-O-glucosides and was involved in defense responses (Kiran et al., 2006).

Given that the complex nature of plant GH1 hydrolases on deglycosylation, our group is thus interested in exploring the molecular mechanisms of this group of enzymes under abiotic stress. By locating the specific substrates (e.g., metabolites or glycosylated proteins), may help us to get a better understanding of their evolutionary divergence and biological significance. Therefore, as an initial step, we constructed a phylogenetic tree using 304 sequences of 14 species in order to reveal the evolution of BGLUs in plant lineages in this study. Subsequently, the spatio-temporal expressions of BGLUs from both Arabidopsis and rice have been studied. Following promoter analysis further demonstrated the transcriptional regulation of Arabidopsis BGLUs is tightly linked to their 5′-flanking regions. At last, the selected Arabidopsis BGLUs which are constitutively induced by salt stress were tested using their T-DNA insertion mutants. These knock-out mutants were less sensitive under sodium chloride (NaCl) treatment, demonstrating the potential roles of AtBGLUs in salt responses. To summarize, our analysis on plant BGLUs using a combination of bioinformatics, molecular and genetic approaches to investigate the evolutionary path, differential transcriptional regulation and potential protein functions of plant BGLUs.

## Materials and methods

### Identification of GH1 hydrolases and sequence alignment

Plant protein sequences consisting of a BGLU domain were obtained by searching nonredundant protein sequences from the NCBI database (National Center for Biotechnology Information, http://blast.ncbi.nlm.nih.gov/Blast.cgi) and Phytozome v11.0 (http://www.phytozome.net/). Fourteen available complete genome sequences selected for the phylogenetic analysis include those from model organisms including chlorophytes (*Chlamydomonas reinhardtii*), bryophyte (*Physcomitrella patens*), lycophyte (*Selaginella moellendorffii*), Arabidopsis (*Arabidopsis thaliana*), grape (*Vitis vinifera*), cucumber (*Cucumis sativus*), papaya (*Carica papaya*), castor bean (*Ricinus communis*), soybean (*Glycine max*), barrel medic (*Medicago truncatula*), sorghum (*Sorghum bicolor*), rice (*Oryza sativa*), maize (*Zea mays*), and cottonwood (*Populus trichocarpa*). There are 382 protein sequences with BGLU domains were obtained. The similarity amongst the sequences of every BGLUs were aligned using software ClustalW2 (http://www.ebi.ac.uk/Tools/msa/clustalw2/). The open gap penalty and extend gap penalty have been set to 10 and 0.2 in multiple alignments, respectively. The accession number or gene identifier, number of sequences and sample accession numbers used in this study are listed in the Table S1. Protein sequences (<30% identity) tested by the similarity matrix were removed before the phylogenetic analysis. A number of 304 sequences were selected and used to construct the evolutionary tree across plant lineages. In addition, 189 sequences of selected model plant and crop species were chosen to build the second phylogenetic tree.

### Phylogenetic and protein structure analysis

The phylogenetic trees were constructed based on the alignment of plant BGLU conserved domains (Figure S1) and generated using neighbor-joining (NJ) methods implemented in MEGA 7.0. The bootstrap values were calculated from 1,000 replicates.

The structural alignments and superimposition from 304 selected BGLU sequences were performed using the input from ClustalW2 based on crystal structure solved in experiments (i.e., PDB ID, 2JF7, 2JF6). The ConSurf Web (http://bental.tau.ac.il/new_ConSurfDB/) was used for the identification of functional protein regions (Ashkenazy et al., 2010). The conservation scores were calculated by using ML approach in ConSurf. Models and figures were drawn using Pymol (DeLano Scientific).

### Multiple Em for motif elicitation (MEME)

The MEME analysis has been carried out using protein sequence of Arabidopsis and rice BGLUs. The MEME server (http://meme-suite.org/tools/meme) was used to calculate the conserved motifs within Arabidopsis BGLUs and rice BGLUs, respectively.

### Plant materials and growth conditions

The knockout mutant of AtBGLU1 (SALK_060948, GABI_341B12) and AtBGLU19 (SALK_007445, GABI_103F10) were obtained from Arabidopsis Biological Resource Center (ABRC) (https://www.arabidopsis.org/). Seeds used for seedling root length assays were surface-sterilized with 20% bleach and 0.05% Tween-20, and then grown on Murashige and Skoog (MS) medium (Murashige and Skoog, 1962) supplemented with 0.8% w/v agar and 1.5% w/v sucrose under the 16 h light (23°C)/8 h dark (21°C) cycles.

### Stress treatments, RNA extraction and quantitative real-time PCR

Arabidopsis (Col-0) were grown on MS plates supplemented with 0.8% w/v agar and 1.5% w/v sucrose for 12 days before treatments. The growth condition for rice seedlings (*Oryza sativa*, Nipponbare) was described previously (Meng et al., 2011). In brief, seeds were germinated in distilled water for 2–4 days at 28°C. Subsequently, 7-d-old seedlings were transferred to a tray and floated in nutrient solution (20 mg/L NH_4_NO_3_, 0.05 mg/L KH_2_PO_4_, 10 mg/L KCl, 1 mg/L MgCl_2_, 1 mg/L FeNaEDTA and trace elements in Hoagland solution) in greenhouse under 16 h light /8 h dark (28°C) cycles. 12-day-old Arabidopsis and 14-day-old rice seedlings were treated at 4°C (16 h light/8 h dark cycles) vs. the room temperature control, 15% (w/v) PEG and 125 and 150 mM NaCl vs. water control. Samples (whole seedlings of Arabidopsis and rice) for RNA extraction were collected at different time points after each treatment. Three plates with randomized position of each time point were pooled together. Each treatment was performed twice.

Plant total RNA was extracted by using the RNeasy Mini Kit (Qiagen, Germany) according to the manufacturer's instruction. Total RNA (5 μg) was reverse-transcribed into cDNA by using Superscript First-Strand Synthesis System (Invitrogen, USA) following the manufacturer's protocol. For both the evaluation of tissue-specific expression and transcript changes under stress treatment, real-time quantitative PCR was performed using FastStart Universal SYBR Green Master (Roche, Germany) in a total volume of 15 μl per reaction on StepOne Plus (Applied Biosystems) (Du et al., 2013). Transcript changes were normalized using Arabidopsis and rice *ACTIN* gene (At3g18780 and Os03g50885) and analyzed from three independent technical replicates as described previously (Czechowski et al., 2005). Each treatment and subsequent RNA extraction and qRT-PCR analysis were performed twice. The gene-specific primer sequences used in this study are shown in Table S2.

### Measurement of primary root length

Measurement of primary root length was carried out as described by Du et al. (2013). Four-day-old seedlings of Col-0, *atbglu1*, and *atbglu19* grown on MS plates were transferred to MS control plates and MS medium supplemented with 125 and 150 mM NaCl. The seedlings were grown vertically under light/dark cycles of 16 h/8 h at 23°C. Photos were taken 10 d after transplantation.

### Analysis of the 5′-flanking regions

The 1.5-kb 5′-flanking regions of Arabidopsis BGLUs was used to identify regulatory motifs. Transcriptional *cis*-elements were predicted using software server PlantCARE (http://bioinformatics.psb.ugent.be/webtools/plantcare/html/) (Lescot et al., 2002).

### Accession numbers

**Figure 1:**
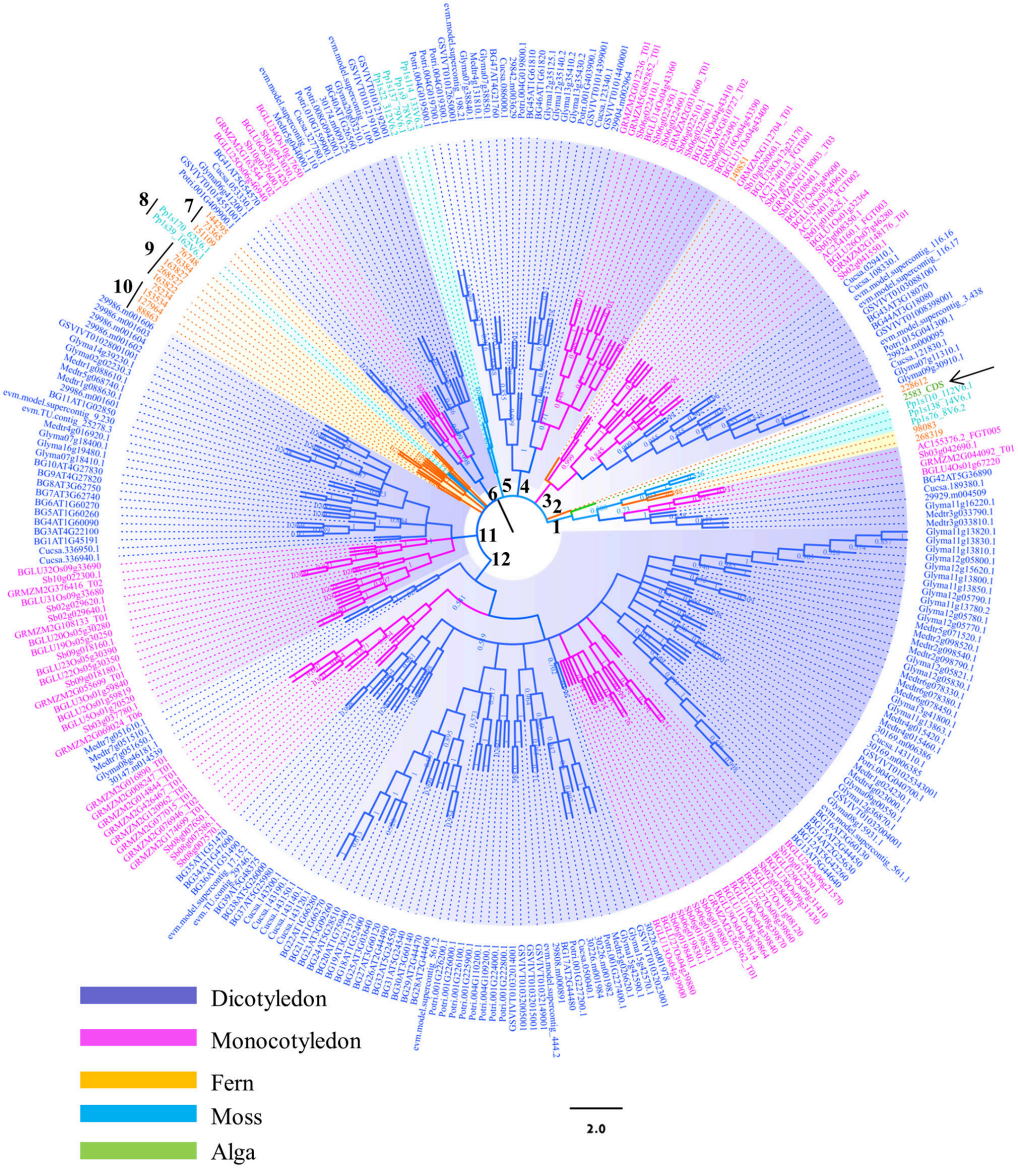
**Phylogenetic analysis of the β-glucosidases in plant lineage**. Phylogenetic analysis of plant β-glucosidases using neighbor-joining (NJ) methods as implemented in MEGA 4.0. Three hundred and four sequences selected from different plant species were used to construct the evolutionary tree. The assigned gene identifiers or accession numbers for the genes are displayed. Bootstrap values are recorded at the nodes. Abbreviations of generic names are listed in the Materials and methods and Table S1.

Sequence data in this article are available in the *Arabidopsis* Genome Initiative and GenBank databases under the accession numbers listed in Figure 1 and Table S1.

## Results

### Identification and phylogenetic analysis of plant GH1 hydrolases

In order to identify the plant GH1 hydrolases, BLASTp searches coupled with conserved domain analysis were conducted using NCBI database and Phytozome v11.0. A total of 382 GH1 hydrolases sequences from 14 plant species were collected and evaluated in terms of similarity. The 304 sequences with certain degree of similarities (greater than 30% amino acid identity) were then used to construct the phylogenetic tree (Table S1). The selected plant species represented different branches on the evolutionary tree for green plant GH1 hydrolases.

The topologies of phylogenetic tree were complicated due to the large number of candidate sequences (Figure 1). Twelve branches were constructed from the inner arch. The bootstrap value (BS) was low at some clades of the tree, implying that the sequences of this gene family were divergence and less reliable. Therefore, analyzing the whole phylogenetic tree may lose information which is significant in evolutionary path. However, the general trend of evolution, from lower plants to higher plants, was similar to other phylogenetic studies (Meng et al., 2011). For example, the green algae (*Ostreococcus lucimarinus*) in branch 1 (pointed by black arrow) is a representative member of lineage that diverged before the evolution of land plants. The GH1 hydrolase sequence of this species showed high possibility that was distantly related to the other plant GH1 hydrolases. Surprisingly, AtBGLU42 and OsBGLU40 was grouped in the same clade with the green algal GH1 hydrolase in branch 1. The long distance of these two GH1 hydrolases to the other members in Arabidopsis and rice may indicate they have different ancestors in comparison to the other Arabidopsis or rice GH1 members. Previous phylogenetic study indicates that OsBGLU40 has 70% identity with bacterial proteins (Xu et al., 2004; Opassiri et al., 2006; Gomez-Anduro et al., 2011; Zhao et al., 2012). They proposed that this gene may have been gene transcripts that were captured by retrotransposons and reincorporated into the rice genome, or by lateral gene transfer from a bacteria species. However, no such results mention AtBGLU42. Further analysis is needed to unravel this clustering. Besides, several other sequences from maize and soybean were also found in this branch (Figure 1), indicating that this gene transfer mechanism may be common amongst higher plants. Mosses, together with the lycophytes, are two early-divergent lineages of land plants. The two species selected in this study, *Physcomitrella patens* and *Selaginella moellendorffii*, were diversely distributed in several clades (Figure 1). For instance, the branches 5 and 8 were clustered by sequences from *Physcomitrella patens*, whereas branches 1, 2, 7, 9, and 10 consisted of sequences from *Selaginella moellendorffii*. They usually formed the basis of each sub-clade together with GH1 hydrolases from other plant species. This phenomenon is similar to the plant kingdom phylogeny, where the protein sequences from these two lower plants built up the basis to the higher plants. However, our analysis indicates that the majority of them were clustered in distinct branches. The GH1 hydrolases from higher plants were not always found to form a monophyletic group. Instead, sequences from the same species were observed to form sub-clades with relatively long evolutionary distance in branches 1, 3, 4, 6, 11, and 12. This phenomenon shown in the phylogenetic analysis may indicate their multi-ancestral origins and diverse functions in plant development and stress responses. Further evidence was obtained using Arabidopsis and rice as a representative dicot and monocot, respectively. The MEME analysis revealed that 2 of 3 motifs identified were not conserved between Arabidopsis BGLUs (Figure S2) and rice BGLUs (Figure S3), suggesting high divergence between the lineages of dicots and monocots.

### Tissue expression pattern in Arabidopsis BGLUs (AtBGLUs) and rice BGLUs (OsBGLUs)

Nowadays, transcript abundance is easily obtained from large-scale microarray and transcriptome analysis using online data mining tools or websites. However, the datasets generated from profiling experiments need to be validated using a second method, such as qRT-PCR. Furthermore, both microarray and RNA sequencing have uncertainties to differentiate transcripts with high similarity, which is the case in BGLU gene family. In addition, the steady state mRNA level will be altered due to a number of factors like growth condition, growth medium composition, sampling time and RNA extraction methods *etc*. Therefore, in order to accurately determine the expression of BGLU genes, primers that can differentiate homologous BGLU genes were designed for the targeted qRT-PCR gene expression analysis (Table S2).

**Figure 2:**
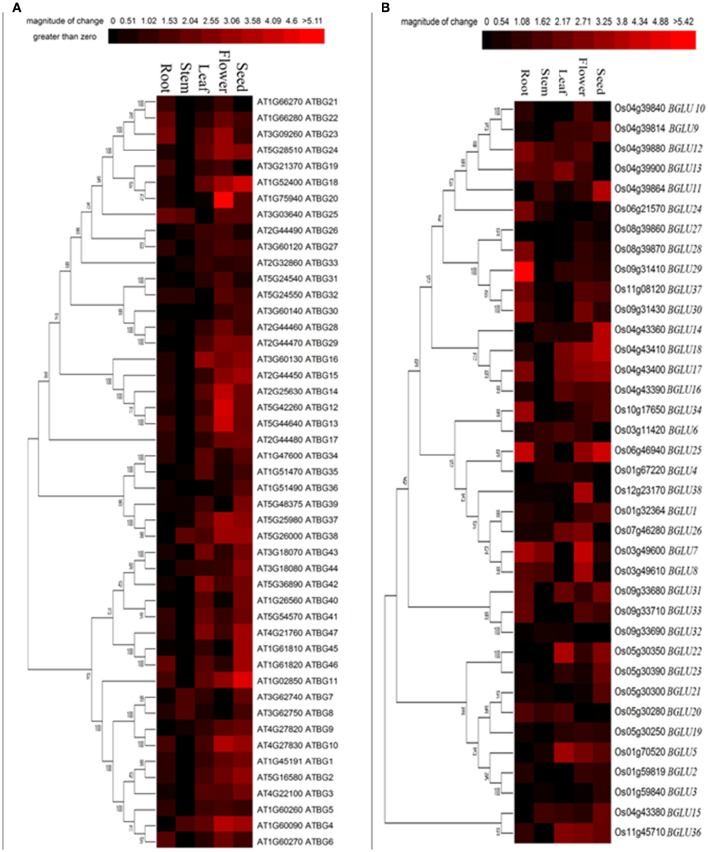
**Tissue-specific expression of Arabidopsis and rice BGLU genes**. Hierarchical cluster analysis was applied to Arabidopsis and rice BGLU genes in different tissue types. The relative gene expression values (log2 scale of qRT-PCR, *n* = 3, technical replicates) were analyzed using the R language programmed heatmap format. Red color represents the level of expression.

To determine the tissue-specific expression pattern of different GH1 hydrolases, we further focused on Arabidopsis and rice sequences, which are representatives of dicots and monocots, respectively. Although some GH1 BGLUs have been demonstrated to be enzymes other than β-glucosidases (Xu et al., 2004), we examined the expression patterns of annotated 47 Arabidopsis (Col-0) *BGLU* genes and 37 rice (Nipponbare) *BGLU* genes in five tissues including seed, flower, leaf, stem and root. As shown in Figure 2, most *AtBGLUs* and *OsBGLUs* genes were expressed in different parts of tissues, among which *AtBGLU6, AtBGLU22, OsBGLU24, OsBGLU28, OsBGLU29*, and *OsBGLU34* were highly expressed in roots than in leaves. Meanwhile, a goodly part of *BGLU* genes such as *AtBGLU10, AtBGLU12, AtBGLU13, AtBGLU14, AtBGLU20, OsBGLU7*, and *OsBGLU26* were found to be highly expressed in flowers. Interestingly, some *BGLUs* specifically expressed in seeds (e.g., *AtBGLU11, AtBGLU18, OsBGLU11, OsBGLU14*, and *OsBGLU25*), suggesting their potential role in seed establishment or germination. The expression of *OsBGLU14* was consistent with previous study, where it was highly expressed in embryo and endosperm (Baiya et al., 2014). In addition, the *BGLUs* classified in the same clades did not always show the co-expression pattern. For example, *OsBGLU32* was classified in the same large clade as *AtBGLU6*. Considerably low expression was detected of *OsBGLU32* in root tissue in comparison to the root transcript level of *AtBGLU6*. Taken together, our results showed that the expression pattern of the both Arabidopsis and rice *BGLU* gens was diverse and had tissue-specific preference.

### *AtBGLUs* and *OsBGLUs* are differentially regulated under cold stress

**Figure 3:**
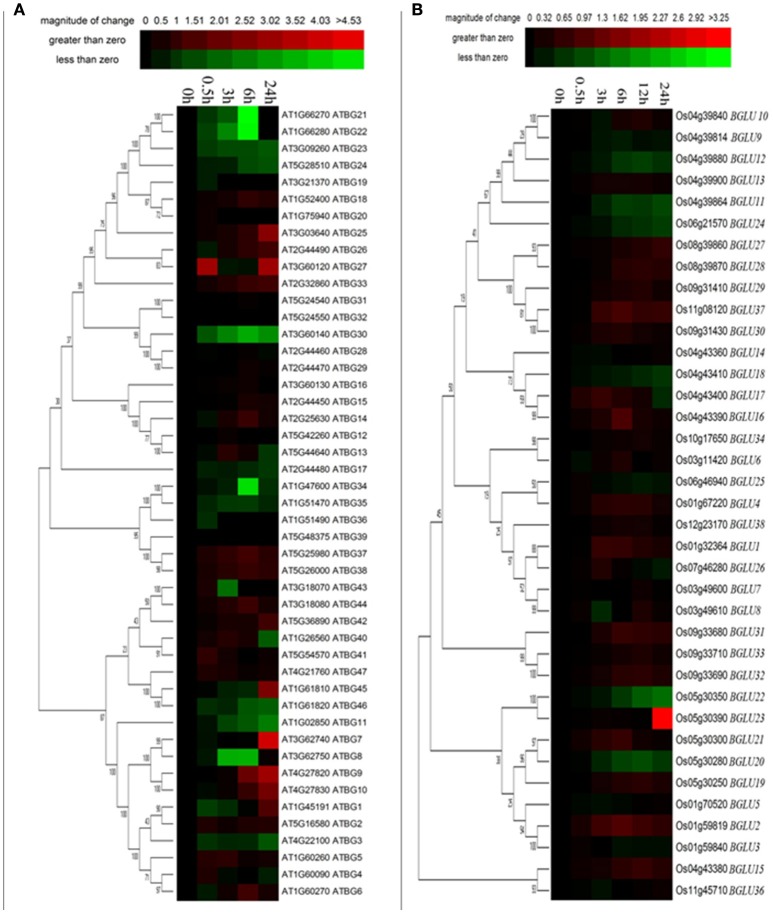
**Expression of Arabidopsis and rice BGLU genes under cold treatment**. Hierarchical cluster analysis were used for the expression of Arabidopsis and rice BGLU genes after **(A)** 0.5, 3, 6, and 24 h or **(B)** 0.5, 3, 6, 12, and 24 h cold treatment. The relative gene expression values (log2 scale of qRT-PCR, *n* = 3, technical replicates) were analyzed using the R language programmed heatmap format. Red and green colors represent the up-regulation or down-regulation of gene expression, respectively.

It has been mentioned in several publications that plant GH1 enzymes play an important role in the cold stress response (Fourrier et al., 2008). Therefore, we investigated the gene expression of both Arabidopsis and rice *BGLUs* in response to a time-course cold treatment. In comparison to rice *BGLUs*, the Arabidopsis *BGLUs* generally showed larger variation in their expression under cold treatment in comparison to the control (Figure 3). Some of *BGLU* genes in Arabidopsis were strongly induced upon the cold stress (Figure 3A). For example, the expression of *AtBGLU7* was significantly up-regulated and the expressions of *AtBGLU11* and *AtBGLU46* were largely down-regulated when the cold treatment lasted for 24 h. Interestingly, very few of *OsBGLUs* were stimulated by the cold stress even at the different time points of cold treatment (4°C) (Figure 3B). This result suggested that OsBGLU family may not play major roles in cold stress responses in rice or the regulation is not at the transcription level. Since *Oryza sativa L*. (Nipponbare) used in this study is a tropical species, it maybe not generally adapted to cold stress.

### Most of *AtBGLUs* and *OsBGLUs* were regulated under PEG treatment

**Figure 4:**
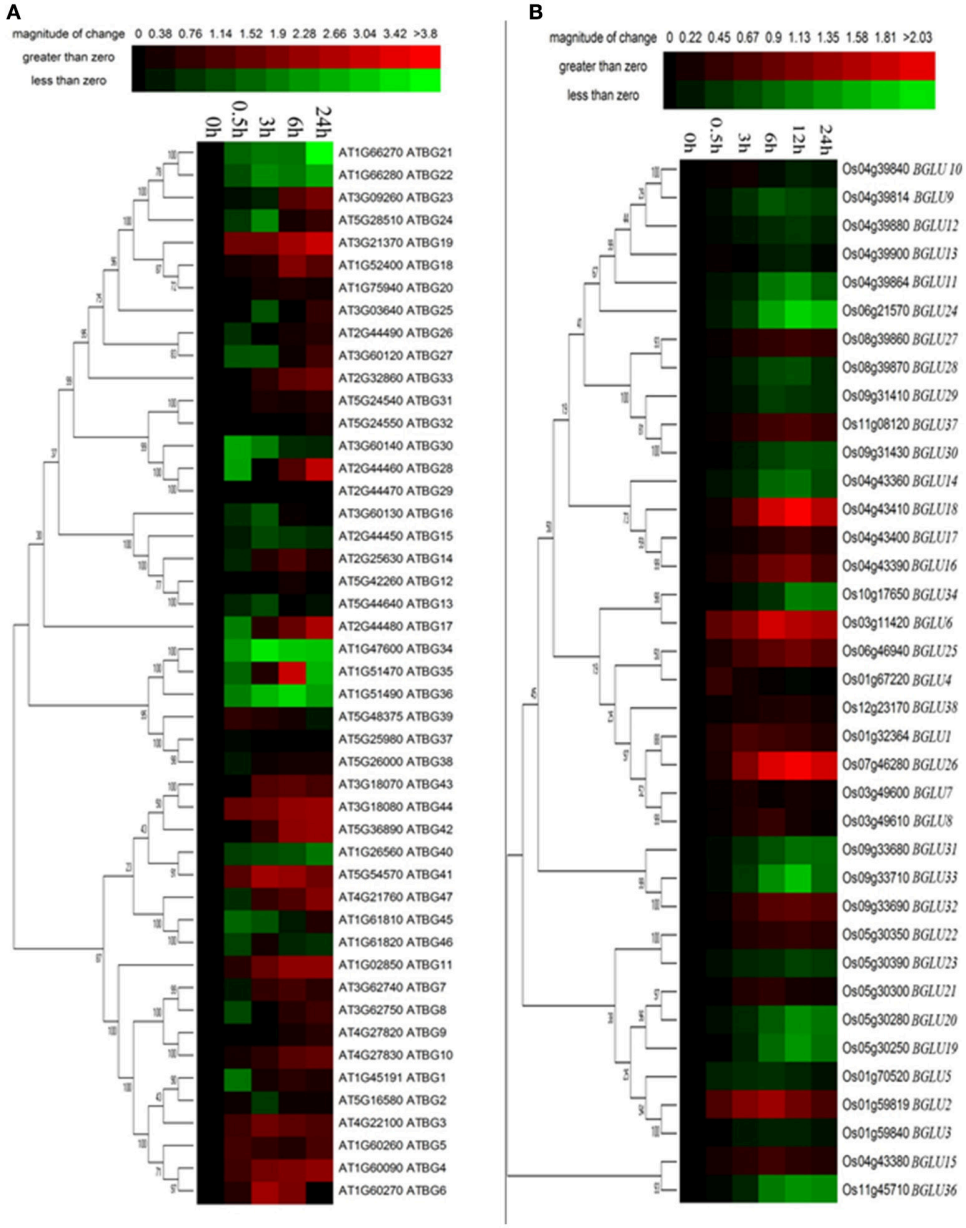
**Expression of Arabidopsis and rice BGLU genes under PEG treatment**. Hierarchical cluster analysis were used for the expression of Arabidopsis and rice BGLU genes after **(A)** 0.5, 3, 6, and 24 h or **(B)** 0.5, 3, 6, 12, and 24 h 15% (w/v) PEG treatment. The relative gene expression values (log2 scale of qRT-PCR, *n* = 3, technical replicates) were analyzed using the R language programmed heatmap format. Red and green colors represent the up-regulation or down-regulation of gene expression, respectively.

Osmotic stress is a representative abiotic stress worldwide. We used 15% PEG treatment to mimic the condition of osmotic stress. It appeared that a total of 16 of Arabidopsis BGLU genes were strongly induced under PEG treatment in comparison to the water control, whereas 11 genes were solely down-regulated (Figure 4A). The rest of the AtBGLUs were either up- or down-regulated in the different time point after PEG treatment. In contrast, most of OsBGLU genes exhibited low expression levels and were not induced or suppressed in early time points (i.e., <3 h) under PEG treatment. At the later time points (i.e., >3 h), the expression of a large number of OsBGLUs were altered (Figure 4B). Therefore, our results showed that the BGLU family genes in Arabidopsis and rice may play distinct roles in drought stress responses.

### Gene expression patterns of *AtBGLUs* and *OsBGLUs* under salt treatment are similar to the PEG treatment

**Figure 5:**
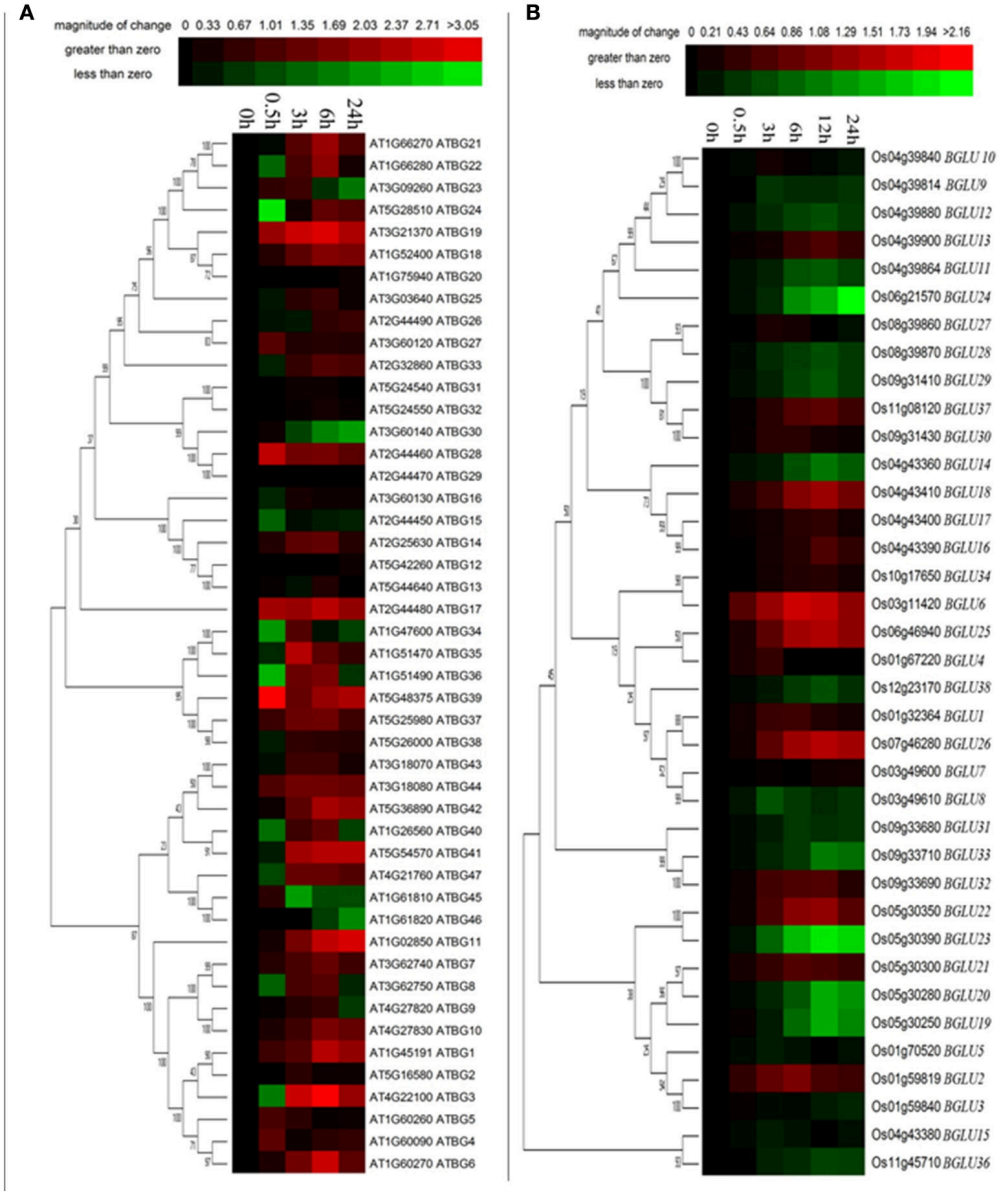
**Expression of Arabidopsis and rice BGLU genes under NaCl treatment**. Hierarchical cluster analysis were used for the expression of Arabidopsis and rice BGLU genes after **(A)** 0.5, 3, 6, and 24 h or **(B)** 0.5, 3, 6, 12, and 24 h 150 mM NaCl treatment. The relative gene expression values (log2 scale of qRT-PCR, *n* = 3, technical replicates) were analyzed using the R language programmed heatmap format. Red and green colors represent the up-regulation or down-regulation of gene expression, respectively.

High salinity is one of the most important stress conditions in many parts of the world, including northern China. To investigate the gene expression changes of *AtBGLUs* and *OsBGLUs* in response to the salt stress, the time course experiment was performed for both Arabidopsis and rice seedlings. Amongst them, large number of AtBGLU family members were up- or down-regulated by NaCl. For example, *AtBGLU3, AtBGLU6, AtBGLU21, AtBGLU22* were significantly induced in the 6-h NaCl-treated samples, but was reduced at the 24 h (Figure 5A) in comparison to the water control. On the other hand, Some AtBGLUs like *AtBGLU30, AtBGLU45*, and *AtBGLU46*, were down-regulated under NaCl treatment (Figure 5A). The AtBGLU45 and 46 were described as monolignol glucosidases in lignification (Escamilla-Trevino et al., 2006), the relationship between these two enzymes and salt stress remains unclear. Differently, *AtBGLU1, AtBGLU11, AtBGLU19*, and *AtBGLU42* were highly up-regulated upon the NaCl treatment, indicating that they may play crucial roles in response to high salinity (Figure 5A). In contrast, the expression level of OsBGLUs were not largely altered until a 6-h treatment by NaCl (Figure 5B), suggesting that they may participate in long term salt responses.

### The Arabidopsis T-DNA mutants of two AtBGLU genes were less sensitive to salt treatment

**Figure 6:**
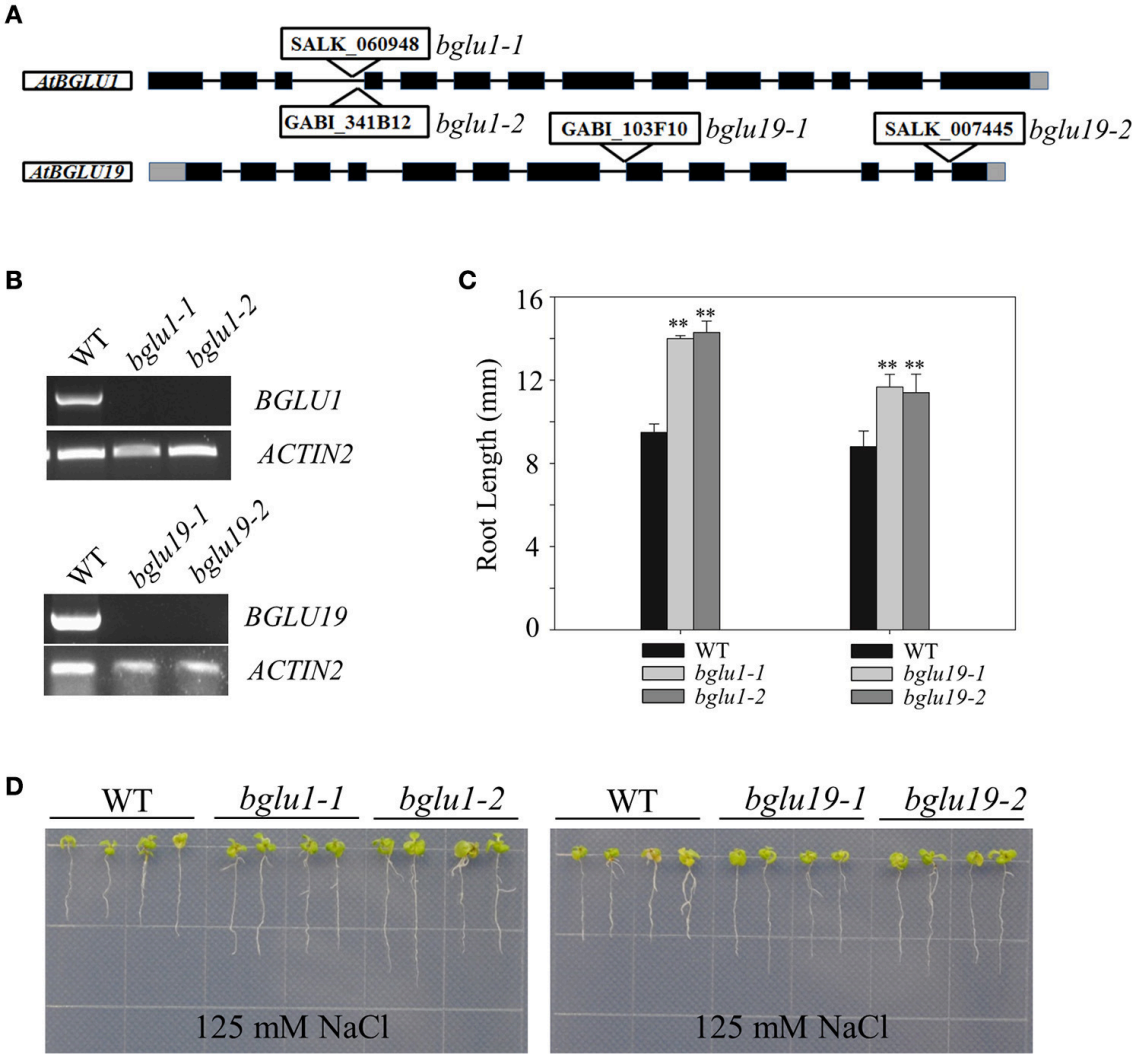
**Phenotypic characterization of AtBGLU1 and AtBGLU19 under NaCl treatment. (A)** Schematic view of T-DNA insertion for *atbglu1* and *atbglu19*. Black and gray boxes are exons and 5′ or 3′-UTR regions, respectively. **(B)** Semi-quantitative reverse transcription polymerase chain reaction (RT-PCR) for identification of Arabidopsis knockout mutants. Plant RNA was extracted from rosette leaves of 4-wk-old WT, *atbglu1*, and *atbglu19* plants. *AtACTIN2* was used as control. **(C)** Relative primary root length amongst WT, *atbglu1*, and *atbglu19* seedlings under NaCl treatment were shown. Values are means ±SE (*n* = 20; 20 seedlings per genotype were measured from 3 plates and the experiments were repeated twice). **(D)** WT, *atbglu1*, and *atbglu19* plants seeds were germinated on MS medium for 4 days, and seedlings were subsequently transferred to MS supplemented with 125 mM NaCl for 10 days. Asterisks indicate significant differences compared with wild type by Student-*T* test (^**^P < 0.01).

The constitutive induction of certain AtBGLUs under salt-treatments draw us attention to perform further analysis. Two knockout T-DNA insertion mutants of *AtBGLU1* (SALK_060948, GABI_341B12) and *AtBGLU19* (SALK_007445, GABI_103F10) were characterized and subjected to NaCl treatment, respectively (Figures 6A,B). Under 125 mM NaCl treatment, both the *atbglu1* and *atbglu19* mutants showed less sensitive phenotypes in comparison to the wild-type plants (Figures 6C,D). Longer primary root length was detected after 7-day growth on the NaCl-containing MS plates (Figures 6C,D), indicating that the constitutive induction of *AtBGLU1* and *AtBGLU19* under NaCl treatment successfully linked the genetic evidence. The potential roles of these two AtBGLUs are being further studied to reveal their molecular mechanisms in response to high salinity.

## Discussion

### Gene structures and genome locations imply the duplication of GH1 hydrolases in higher plants during their evolution

**Figure 7:**
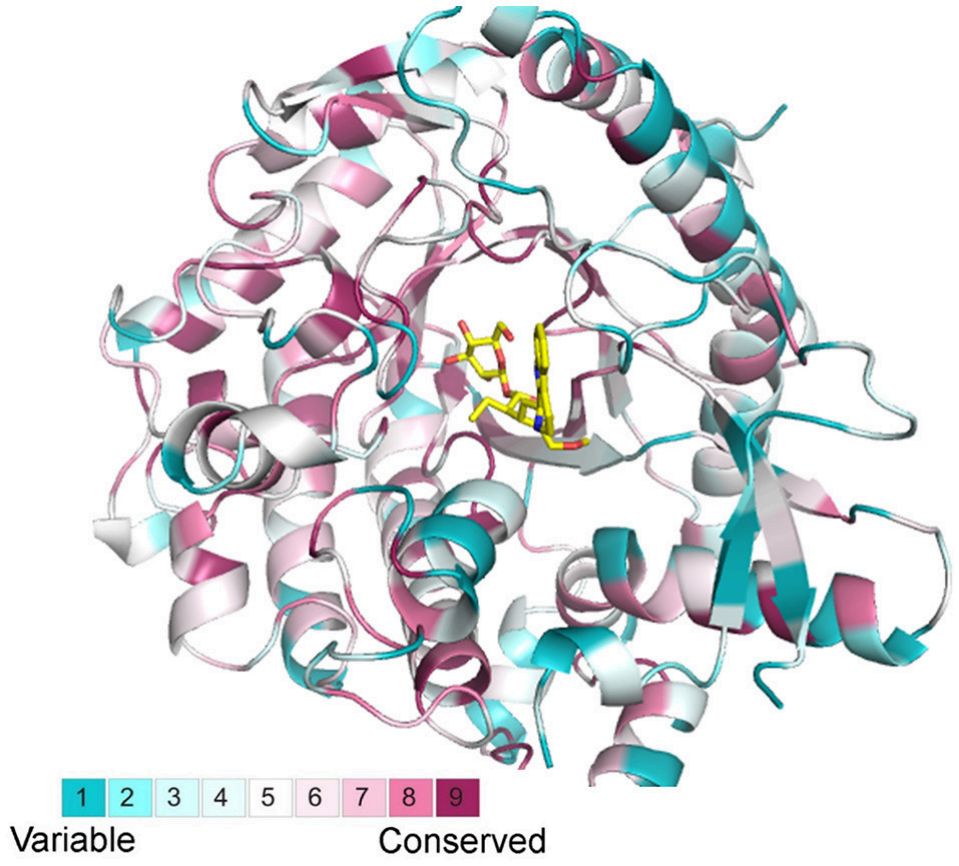
**Structural alignments from selected BGLU homologs with the canonical BGLU structure**. Ribbon representations of the BGLU structure are colored according to conservation in 304 selected BGLU sequences from phylogenetic analysis, the color representation blue to red indicates the increasing of conservation.

The output from BLASTp search for GH1 hydrolases revealed their complexity in gene structures and sequence homology (Figure 1). Eighty amino acid residues show over 90% conservation amongst 304 aligned sequences and were represented in a structure model based on known β-glucosidase structure information (Figure 7). Besides, the other parts of the protein sequences were diverse, even at the place of enzyme active site pockets, suggesting their potential ability to use a large spectrum of substrates. The selected proteins, ranging from 315 to 672 amino acids, were used to construct the evolutionary tree (Figure 1). Although the degree of similarity of some clades was low, some features were observed to reveal their evolutionary relationships. For instance, the presence of multiple homologous members in both early-divergent plant lineages and higher plants demonstrated that the function of GH1 hydrolases evolved before the evolution of land plants (Figure 1). Furthermore, gene duplication was a common feature across the lineages. The coexistence of multifold GH1 hydrolases amongst the plants may indicate their crucial roles in plant survival (Figure 1). Some researchers have studied the phylogenetic relationships within one species or several species belong to the higher plants (Xu et al., 2004; Opassiri et al., 2006; Gomez-Anduro et al., 2011; Zhao et al., 2012). No existing publications have mentioned the evolutionary relationships across the plant lineages (i.e., from lower plants to higher plants), maybe due to the sequence divergence of this gene family. From our results, the divergence of plant GH1 hydrolases started approximately at the stage between the evolution of mosses and lycophytes in comparison to the green algae (Figure 1), suggesting that the evolvement of neo-functionality in this family started as early in lower plants.

### Promoter analysis of *AtBGLUs* correlates their expression data in response to abiotic stresses

Regulatory promoters have been considered to be one of the most important factors in regulating gene expression (Chen et al., 2014b). They often located in the few hundred base pairs before the transcription start site of a gene. To further explain the relationships between the gene expression and genetic phenotypes in this study, the 1.5-kb 5′-flanking regions of all the AtBGLUs were used to analyze the presence of stress-responsive *cis*-elements. The detailed results were listed in Table 1. Multiple *cis*-elements in response to temperature and drought stresses have been detected by software PlantCARE (Lescot et al., 2002), verifying that the regulation of the gene expression largely correlates to the motifs found in the promoter regions of AtBGLUs. For instance, LTR motifs have been reported to be regarded as responsive *cis*-element under low temperature (Hughes and Dunn, 1996). The expression of *AtBGLU9* was up-regulated at 6 and 24 h after the cold treatment. The three LTR motifs located in the 5′-flanking region of this gene served as good candidates to this regulation (Table 1). Interestingly, the *AtBGLU19*, which was identified to be involved in salt stress response, had several ABA-responsive elements in their promoter regions such as G-box and ABA-responsive elements (ABRE) (Table 1). Abscisic acid constitutes one of the major pathways in response to salt stress (Chinnusamy et al., 2004; Hirayama and Shinozaki, 2010). Transcriptional regulation of ABA-responsive genes converges to transcription factors. To date, various transcription factors from different families in Arabidopsis have been reported to function in ABA-mediated gene activation or repression (Fujita et al., 2013). Both G-box and ABRE are candidate sequences for the binding of ABA-RESPONSIVE ELEMENT BINDING PROTEINS/ABA-RESPONSIVE ELEMENT BINDING FACTORS (AREB/ABFs) (Choi et al., 2000; Uno et al., 2000). This group of transcription factors belongs to the core module of ABA signal transduction. They belong to the basic leucine zipper (bZIP) transcription factor family (Jakoby et al., 2002) and use G-box type ABRE with a consensus ACGT core. Two independent groups isolated several members of this family through yeast-one hybrid screening (Choi et al., 2000; Uno et al., 2000). From phylogenetic analysis, all the ABA-responsive bZIP transcription factors belong to Group A bZIPs (Jakoby et al., 2002). Several transcription factors are well documented including AREB1/AtABF2, AREB2/AtABF4, ABF3, and ABI5 amongst the 13 members in this group (Brocard et al., 2002; Carles et al., 2002; Kim et al., 2004; Fujita et al., 2005; Yoshida et al., 2010). The high density of this type of motifs found in the promoters of *AtBGLU19* indicates the possibility that the induction of these two genes under NaCl treatment results from the ABA-responsive core regulation. In contrast, only one G-box was found in the promoter of *AtBGLU1* (Table 1), suggesting that other unknown motifs may participate in salt responses to drive its expression.

**Table 1:** **List of stress-responsive elements located in the 1.5-kb 5′-flanking regions of Arabidopsis BGLUs**.

**AtBGLUs**	**Locus**	**Presence of stress-responsive *cis*-elements**	**Total**
AtBGLU1	At1g45191	G-box (2)	2
AtBGLU2	At5g16580	ARE (1), G-box (2), HSE (1)	4
AtBGLU3	At4g22100	ABRE (1), ARE (1), G-box (2), GARE (1)	5
AtBGLU4	At1g60090	ARE (2), GARE (2), HSE (2)	6
AtBGLU5	At1g60260	ARE (1), ERE (1), HSE (1)	3
AtBGLU6	At1g60270	ABRE (1), ARE (1), ERE (1), G-box (2), HSE (1)	6
AtBGLU7	At3g62740	ARE (1), G-box (4), GARE (3), HSE (1), P-box (1)	10
AtBGLU8	At3g62750	ABRE (2), ARE (4), G-box (2), GARE (1), HSE (1), LTR (1), AP-2 like (1)	12
AtBGLU9	At4g27820	AREB (1), ARE (3), LTR (3)	7
AtBGLU10	At4g27830	ARE (2), G-box (1), GARE (1), HSE (1), LTR (1), P-box (2)	8
AtBGLU11	At1g02850	ARE (2), G-box (2), GARE (2), P-box (2)	8
AtBGLU12	AT5G42260	ABRE (2), ARE (1), G-box (5), GARE (1), HSE (3)	12
AtBGLU13	AT5G44640	ABRE (1), ARE (5), G-box (3), GARE (1), LTR (1)	11
AtBGLU14	AT2G25630	ABRE (3), ARE (2), CE (1), ERE (2), G-box (8), LTR (1)	17
AtBGLU15	AT2G44450	ARE (1), ERE (1), G-box (11), HSE (1)	15
AtBGLU16	AT3G60130	ARE (3), ERE (2), G-box (4), HSE (2), P-box (1), AP-2 like (1)	13
AtBGLU17	AT2G44480	ABRE1 (1), ARE (5), G-box (5), TATC-box (1)	12
AtBGLU18	AT1G52400	ABRE (1), ARE (1), ERE (1), G-box (6), HSE (3)	12
AtBGLU19	AT3G21370	ABRE (4), ERE (1), G-box (6), P-box (2)	13
AtBGLU20	AT1G75940	ABRE (3), ARE (1), G-box (8), GARE (2), HSE (1), LTR (1)	16
AtBGLU21	AT1G66270	ARE (7), ERE (1), G-box (5), HSE (1), LTR (1)	15
AtBGLU22	AT1G66280	ABRE (2), ARE (2), G-box (4), GARE (1), LTR (1), TATC-box (1)	11
AtBGLU23	AT3G09260	ARE (1), G-box (2)	3
AtBGLU24	AT5G28510	ABRE (1), ARE (1), G-box (2), HSE (2)	6
AtBGLU25	AT3G03640	ARE (2), G-box (5), GARE (1), HSE (1)	9
AtBGLU26	AT2G44490	ABRE (2), ARE (2), G-box (4), GARE (1), HSE (2)	11
AtBGLU27	AT3G60120	ARE (2), G-box (1), HSE (1)	4
AtBGLU28	AT2G44460	ABRE (4), ARE (2), ERE (1), G-box (6)	13
AtBGLU29	AT2G44470	ABRE (1), ARE (1), G-box (6), HSE (2), P-box (1)	11
AtBGLU30	AT3G60140	ABRE (1), ERE (2), G-box (2), LTR (1)	6
AtBGLU31	AT5G24540	ARE (2), G-box (4), HSE (1)	7
AtBGLU32	AT5G24550	ABRE (2), ARE (4), G-box (8), GARE (1), P-box (1)	16
AtBGLU33	AT2G32860	ARE (2), G-box (4), GARE (1), HSE (2)	9
AtBGLU34	AT1G47600	ARE (3), G-box (1)	4
AtBGLU35	AT1G51470	ABRE (2), ARE (1), G-box (1), HSE (1)	5
AtBGLU36	AT1G51490	ABRE (1), ARE (4), G-box (4)	9
AtBGLU37	AT5G25980	ABRE (2), ARE (1), G-box (8), HSE (2)	13
AtBGLU38	AT5G26000	ARE (5), GARE (1), HSE (3), LTR (2), P-box (1)	12
AtBGLU39	AT5G48375	ABRE (2), ARE (1), G-box (3), GARE (3), LTR (2), P-box (2), TATC-box (1)	14
AtBGLU40	AT1G26560	ARE (2), HSE (3)	5
AtBGLU41	AT5G54570	ABRE (1), G-box (4), GARE (1), HSE (1), LTR (2)	9
AtBGLU42	AT5G36890	ARE (2), G-box (1), GARE (1), LTR (2)	6
AtBGLU43	AT3G18070	ABRE (2), ARE (1), G-box (5), GARE (4)	12
AtBGLU44	AT3G18080	ABRE (1), ARE (1), ERE (1), G-box (7), HSE (1), P-box (2)	13
AtBGLU45	AT1G61810	ARE (3), G-box (2), HSE (2), LTR (1), TATC-box (1)	9
AtBGLU46	AT1G61820	ABRE (3), ARE (5), G-box (7), GARE (1), HSE (2)	18
AtBGLU47	AT4G21760	ARE (2), ERE (1), GARE (2), LTR (3)	8

*G-box (CACGTT, CACGAC; ABA), ARE (TGGTTT; Anaerobic), HSE (AAAAAATTTC; Heat stress), ABRE (ACGTGGC; ABA), GARE (AAACAGA; GA); ERE (ATTTCAAA; Ethylene), P-box (CCTTTTG; GA), LTR (CCGAAA; Low temperature), AP-2 like (CGACCAGG; ABA), CE (GACGCGTGTC; ABA), TATC-box (TATCCCA; GA)*.

Besides G-box and ABREs, some other *cis*-elements like P-box (Itoh et al., 1995), TATC-box (Gubler et al., 1995) and GARE (Sutliff et al., 1993) for gibberellins signal transduction, ERE for ethylene signaling pathways (Itzhaki and Woodson, 1993), HSE for heat-shock responses (Gurley and Key, 1991) and ARE for anaerobic induction (Walker et al., 1987) have been observed, indicating that multiple stress responses can alter the gene expression of *AtBGLUs*.

### Importance of plant GH1 hydrolases in plant engineering to tolerate abiotic stresses

In comparison to the biotic stress, abiotic stress showed more difficulties in the application of plant engineering (Wang et al., 2003; Bressan et al., 2009). Transformation of a single gene to target plants is difficult to enhance their abiotic resistance (Chen et al., 2014a). However, abiotic stresses such as low temperature, low water supply and high salinity, share some common secondary stress conditions like the adjustment of osmotic potential (Hirayama and Shinozaki, 2010; Mittler and Blumwald, 2010). Given these phenomenon, engineering effector proteins involved in cross-talk pathways may be beneficial.

Plant GH1 hydrolases are considered to be an ideal target for plant engineering (McManus and Osborne, 1991) since they are involved in different developmental processes and various stress responses by deglycosylation of targeted metabolites or proteins (Xu et al., 2004; Opassiri et al., 2006). In this study, experimental and bioinformatic analysis indicates that both rice and Arabidopsis BGLUs were involved in multiple abiotic stress responses. Thus, using GH1 hydrolases as engineering materials, we can achieve various goals such as manipulation of active metabolite homeostasis, enzyme subcellular localization or activity etc. However, the relatively few functional studies have been reported may due to several lines of difficulties in molecular biology and plant genetics. For example, the number of genes in this family is large and some of the members are highly conserved, giving high degree of redundancy. In addition, the spectrum of substrates is diverse and most of the potential substrates are not commercially available. Thus, the works to determine the substrate specificity for each GH1 hydrolase are time-consuming and technically difficult. Furthermore, some GH1 hydrolases locate in the vicinity of each other in plant chromosome, bringing difficulties for plant double mutant generation by conventional crossing. Therefore, the development of high-throughput technology to screen the substrates for GH1 hydrolases is necessary (Wei et al., 2015). The progress in plant mutant generation using CRISPR-Cas9 technologies (Miao et al., 2013) have served a possible solution on the generation of double or multiple mutants for the members of this gene family.

In conclusion, the evolution and expression of plant GH1 hydrolases have been studied in this research article. The phylogenetic analysis revealed gene duplication may be one of the most important factors in the evolution of GH1 hydrolases across plant lineages. Although some family members of AtBGLUs and OsBGLUs share high similarity, the tissue and stress-responsive expression patterns are diverse. Therefore, their distinct roles in plant development and stress responses need further investigation.

## Author contributions

Y-YC, JZ, and Y-GL designed research scheme. Y-YC, J-FY, T-YL, ZF, F-YZ, Z-FS, TF, and L-JW performed experiments. M-XC, N-HY, and G-FH analyzed data. Y-YC, JZ, Y-GL, and M-XC wrote and revised the manuscript.

### Conflict of interest statement

The authors declare that the research was conducted in the absence of any commercial or financial relationships that could be construed as a potential conflict of interest.
