# Supplementary material for: A Phylogenetically Informed Comparison of GH1 Hydrolases between Arabidopsis and Rice Response to Stressors
Source: Front Plant Sci. 2017 Mar 24;8:350. doi: 10.3389/fpls.2017.00350 (PMC5364172; doi:10.3389/fpls.2017.00350)
Supplement: Supplementary file 1 [file Table1.DOCX]

**Supplementary Table** **1** Sequence summary on phylogenetic analysis.

| Plant species | Common name | No. of BGLUs | Sequence initial *e.g.* |
| --- | --- | --- | --- |
| ***Arabidopsis thaliana*** | **Arabidopsis** | **47** | **AT1G45191** |
| *Carica papaya* | Papaya | 16 | evm.TU.contig_25278.3 |
| *Cucumis sativus* | Cucumber | 23 | Cucsa.029410.1 |
| ***Glycine max*** | **Soybean** | **48** | **Glyma01g06980.1** |
| *Medicago truncatula* | Barrel medic | 28 | Medtr1g024230.1 |
| ***Oryza sativa*** | **Rice** | **38** | **Os01g70520** |
| *Ostreococcus lucimarinus* | Green algae | 1 | 2583 CDS |
| *Physcomitrella patens* | Moss | 16 | Pp1s9_78V6.3 |
| *Populus trichocarpa* | Cottonwood | 27 | Potri.001G222800.1 |
| ***Ricinus communis*** | **Castor bean** | **25** | **30076.m004675** |
| *Selaginella moellendorffii* | Spikemoss | 22 | 73365 |
| ***Sorghum bicolor*** | **Sorghum** | **36** | **Sb01g010840.1** |
| *Vitis vinifera* | Grape | 21 | GSVIVT01008398001 |
| ***Zea mays*** | **Maize** | **36** | **AC148152.3_FGT008**  **GRMZM5G882852_T01** |

The species of plant lineage were listed and the number of BGLUs choosen was mentioned. The species with bold character were selected for further analysis in Figure 1.

**Supplementary Table** **2** Primers used in this article.

| Primer No. | Seqeunce 5’ 🡪 3’ | Remarks |
| --- | --- | --- |
| JHP0113 | AACACGAGCCAAAATTGACAC | T-DNA mutant  SALK_060948 |
| JHP0114 | TGTATGTAAGCCAGTTTCGGC | T-DNA mutant  SALK_060948 |
| JHP0143 | GATTTACCAAAGCCCAAAAGG | T-DNA mutant  SALK_007445 |
| JHP0144 | AAACCAAAGACAAACTCGTCG | T-DNA mutant  SALK_007445 |
| JHP0550 | ATGGAAGATGTTTTGACTCTCATTACC | AtBGLU1-RT |
| JHP0551 | GTTGGCTCTTTTCGTTGCGATTTC | AtBGLU1-RT |
| JHP0552 | GTCAAGATGGACGGTCAGGATTC | AtBGLU19-RT |
| JHP0553 | TCAGAGCTCCTCGTGTAGCTTTG | AtBGLU19-RT |
| At1g45192QRTF | CAGTAGCGATCTTTACAGCAGG |  |
| At1g45192QRTR | GTGAAGGAAAGTATCCCAGACG |  |
| At5g16580QRTF | TCGTTGTTCTAACTGCTCGTC |  |
| At5g16580QRTR | AACCTCCTTGCTTATCCTTGT |  |
| At4g22100QRTF | AGGAGGTTCTGTGGGATTTAGC |  |
| At4g22100QRTR | CTTTCATTTCATCGGGATAGTCA |  |
| At1g60090QRTF | TGGTGGACACTAACTTAGATGCTT |  |
| At1g60090QRTR | CGTGGCTTACTAACTCTTGGATG |  |
| At1g60260QRTF | ACCTCATCAGCGAATTAATAACCC |  |
| At1g60260QRTR | GCCTCATTGATAGTGGTCCAGAAT |  |
| At1g60270QRTF | CGATAGGGTTTAGTCCTTCCACA |  |
| At1g60270QRTR | GTCAGATGAGCCTTTCACTTGTTC |  |
| At3g62740QRTF | TCTTCGGAGCCGCCACTTCT |  |
| At3g62740QRTR | GCCCATTTCTGCCATCAACA |  |
| At3g62750QRTF | CTTACATTGGTGCTGTCCTCAAC |  |
| At3g62750QRTR | CAAATCTATCAGCGACCATACGA |  |
| At4g27820QRTF | TTCTATGTCACAAACCACCAACC |  |
| At4g27820QRTR | ACCATCGGCATACCATTTTCAA |  |
| At4g27830QRTF | TTATGTCACAAACAAACCCTCAC |  |
| At4g27830QRTR | CAAGAATACCTTCAAGACCCCA |  |
| At1g02850QRTF | CTTTAACCGCAGTTTCGTCG |  |
| At1g02850QRTR | GTCAGCAGCTCCTTCCACCT |  |
| At5g42260QRTF | GCGGTCGCTTACCTACATTTACTGC |  |
| At5g42260QRTR | TGATGCTGCCTTTGGACCGATAG |  |
| At5g44640QRTF | CTTTACAAGGAAGATGTGGGTTTA |  |
| At5g44640QRTR | AATGGTGGCGAATGGCTTAA |  |
| At2g25630QRTF | ACACGGTCAGGTTTGGACTT |  |
| At2g25630QRTR | CTGAACCAGTGAGCCGATTT |  |
| At2g44450QRTF | GTGGGAATCTAAAAGGAGGAATCAA |  |
| At2g44450QRTR | CAATGTCATCCAATGCTTCACTCTA |  |
| At3g60130QRTF | CAGCATGGCACTACCCTTAT |  |
| At3g60130QRTR | TTGAGCATTTCGGACTCTTC |  |
| At2g44480QRTF | CTTAATCCTTTCTCATGCCGC |  |
| At2g44480QRTR | CGAAGAAGAAGTCAAGAGCCC |  |
| At1g52400QRTF | TCATAACGCTGATGTTGCTG |  |
| At1g52400QRTR | AATTGGACTCCCACTTTGCT |  |
| At3g21370QRTF | TGTCAAGATGGACGGTCAGGATT |  |
| At3g21370QRTR | ACCAAGCTGGACTATGAGCGATT |  |
| At1g75940QRTF | GGACTGGGAACCAAGGTATG |  |
| At1g75940QRTR | ATGATTTCAGGATTGCCGTA |  |
| At1g66270QRTF | GCCGTTGATTTCTTCCATCGTTA |  |
| At1g66270QRTR | GCTTGACTCACTCCCTTCTCCTTTC |  |
| At1g66280QRTF | CCATTGGTAGCCAGCCTCTT |  |
| At1g66280QRTR | TAATTTCCGGGTTTGCGTAT |  |
| At3g09260QRTF | TTTTCTCGCACGCTGGCTAT |  |
| At3g09260QRTR | ATTCCGATCTTCCCACCTTT |  |
| At5g28510QRTF | CTATTGGGCTGCTTTGGCTAC |  |
| At5g28510QRTR | GTCCACGACAAGTTTCATTCAC |  |
| At3g03640QRTF | GATTACCATCTTTTACGCCCGAGCA |  |
| At3g03640QRTR | CGCAGTAGCAGGCTGAGAACCAA |  |
| At2g44490QRTF | CACGCAGAAGCAGTGGAAGT |  |
| At2g44490QRTR | TGGCTCGTACCAAAGTGGATTA |  |
| At3g60120QRTF | CATCATTTACCGCAGCACAA |  |
| At3g60120QRTR | CCATTCCGAACCACCTCTTA |  |
| At2g44460QRTF | GTGTCAAGGTGGCGATTCAG |  |
| At2g44460QRTR | GTCGGCAGGAGAAGCAGAGT |  |
| At2g44470QRTF | CGAAATGGGTGAATAGTCGGTGTC |  |
| At2g44470QRTR | GCCACCAAGGAGATATCACTATCCC |  |
| At3g60140QRTF | CGTGGTTCTTCATTATCCTTTTT |  |
| At3g60140QRTR | TTCACTTGTTGCACCTTCGTAC |  |
| At5g24540QRTF | GAGCCTTATTTAGTCTCCCACC |  |
| At5g24540QRTR | ATCAGCATTAGAAGCACTGTCAT |  |
| At5g24550QRTF | TCGCTTCACGACTGACCAACACCTC |  |
| At5g24550QRTR | TTCTGATGAGATGTGGTCGCCGC |  |
| At2g32860QRTF | AAAGGTAAGAAAGCACCAGGAC |  |
| At2g32860QRTR | AGCAGCAAGATGAGCAAGAAT |  |
| At1g47600QRTF | GTTGTGGCTCCTAGCTTCGT |  |
| At1g47600QRTR | GAGATGGCTGCAATGGTTTT |  |
| At1g51470QRTF | GGACGAGAATGGGATAACATACTAC |  |
| At1g51470QRTR | AATAGAAGCTCAGCGTAGTTTGTG |  |
| At1g51490QRTF | ATCCACCAGGATTCCGTCAG |  |
| At1g51490QRTR | CGTCCATTATCGGCAAGAGC |  |
| At5g25980QRTF | GCTTGGAGTTAAAGGCTACAGA |  |
| At5g25980QRTR | AAGGTCCCAGTGAAAGAGTGTA |  |
| At5g26000QRTF | GCAGCCAGTTACTACTACCCAA |  |
| At5g26000QRTR | TAGCCTTCTCAAAGTCCTCATC |  |
| At5g48375QRTF | GTTTCAAGATATTCGGTGATAGGGT |  |
| At5g48375QRTR | GGTTATGTGCAACGATATAAGGTTC |  |
| At1g26560QRTF | TTAACTCACGCCACAGTATCCG |  |
| At1g26560QRTR | AAAACCAGCCAAGTTGAAAGTCT |  |
| At5g54570QRTF | AGTCATCGGTCGAACCGTAT |  |
| At5g54570QRTR | GAGCTGCATCTTTGTCCTCA |  |
| At5g36890QRTF | GTCGCCACTTCCGCTTACCA |  |
| At5g36890QRTR | CCACAGCAACATCGCCGTTA |  |
| At3g18070QRTF | TATGGCAACCCAACTATGATTC |  |
| At3g18070QRTR | GCTCCATCGTCTACTGCTTTT |  |
| At3g18080QRTF | TAGGTCCAAGGGCTTACTCGTC |  |
| At3g18080QRTR | GATTCTCGTGGTGTCATGTAGTCC |  |
| At1g61810QRTF | GACCGGAAAGGCAATGTTAC |  |
| At1g61810QRTR | ATCTTGTGGAATCCTGTGGG |  |
| At1g61820QRTF | CGAGCAATGAAATGAACAGC |  |
| At1g61820QRTR | CTCCGTCTCCAGAATTACAAGC |  |
| At4g21760QRTF | TGGCTCTGCTTCATCATCAC |  |
| At4g21760QRTR | TCCCAGTTGCTTAGGGTTTT |  |
| Os01g32364QRTF | TGCCCATCACCTCATTCTTTC |  |
| Os01g32364QRTR | TGCAGCGATTTAGGGTATTCA |  |
| Os01g59819QRTF | ACCCATTGGTATTTGGAGATTACC |  |
| Os01g59819QRTR | AGCACCCTTGATTAGTTCAGTTTG |  |
| Os01g59840QRTF | ACCTCGGCGTACCAGTATGAG |  |
| Os01g59840QRTR | TCCACTTGGGATAAGCCTTGA |  |
| Os01g67220QRTF | AGTTTGGGTTTCCGTGCTTAT |  |
| Os01g67220QRTR | TTGCGTATGGCTCAATACCTT |  |
| Os01g70520QRTF | CCATTGCCGTCGTCTACCTCT |  |
| Os01g70520QRTR | GCTTGGGCTCCTACCATCCTC |  |
| Os03g11420QRTF | GGGATGAGGAGCCTGATGAAC |  |
| Os03g11420QRTR | GGAAGCAGCCAGATTGGTGAG |  |
| Os03g20710QRTF | GCATGTTGGGTTTGTCTATGAA |  |
| Os03g20710QRTR | ATATTGAAGTTGCCAGGTTGGT |  |
| Os03g49600QRTF | GTTGCAGGAAATCAGAATGGAGA |  |
| Os03g49600QRTR | CAAGAGGGAGATCGTAGTGGTAAAG |  |
| Os03g49610QRTF | CTGAACCACTACGATCTCCCACT |  |
| Os03g49610QRTR | GCTGCTACTATCCTTGGCTCATT |  |
| Os04g39814QRTF | ACACCTCAGGCACTGGAAGATA |  |
| Os04g39814QRTR | GTTTCACTCGGTCACCAAACTC |  |
| Os04g39840QRTF | GATTACTGGTAGTCGAAACGGGAT |  |
| Os04g39840QRTR | GGCGAGAAGGTGCTTGTGATAG |  |
| Os04g39864QRTF | TACTATCACAAGCACCTTCTCGC |  |
| Os04g39864QRTR | TTCCGTCATCGTAATCCACAAA |  |
| Os04g39880QRTF | GTGGAGTCAACAAAGAAGGCATAA |  |
| Os04g39880QRTR | TGAGGTGAGTCCCAGTGGAAAA |  |
| Os04g39900QRTF | TCGCCACAGGCATTAGAAGATA |  |
| Os04g39900QRTR | AGCTCAAAGGCTCATTGAAGGT |  |
| Os04g43360QRTF | ACTCCTTGGGTGTCAACTCGTA |  |
| Os04g43360QRTR | GGCATATTGTAACAAATGGCTGT |  |
| Os04g43380QRTF | CGGGAAACCTACTGCATTTTCA |  |
| Os04g43380QRTR | CAAATCTTCCATGTTGGTATCGC |  |
| Os04g43390QRTF | GGAGGAGTTTGAGTACTACTCGGACG |  |
| Os04g43390QRTR | AATGTACTGGTGCCTGGTGGACA |  |
| Os04g43400QRTF | GTCGATCCTGGACGAATCTCAGA |  |
| Os04g43400QRTR | TCAGACGGTTGTAGAAGGCGATT |  |
| Os04g43410QRTF | GGCTACATGCTCGGCACCTAC |  |
| Os04g43410QRTR | AATCATTCCTCTCTGCTTGCTCTG |  |
| Os05g30250QRTF | ATCCGCTGCTCCTCTTCTTCTC |  |
| Os05g30250QRTR | ATGCCACATCACCTGTTTCTCC |  |
| Os05g30280QRTF | CCACGGTTGAGCCATACTTGTT |  |
| Os05g30280QRTR | TATGCCGATAATGCCCTTCTGT |  |
| Os05g30300QRTF | GGCACCGTTACAGGACTTGACT |  |
| Os05g30300QRTF | CCACATCATCCAGGGTTTCACT |  |
| Os05g30350QRTF | CAAGCATCTGGGACACTTTCACT |  |
| Os05g30350QRTR | GAATAAGCCTAGACCAAGAAATGGA |  |
| Os05g30390QRTF | AGGAGGATGTTAAGTTGATGAGTGA |  |
| Os05g30390QRTR | ATCTTGAAGAGCTTGAGGGAAAT |  |
| Os06g21570QRTF | CTCATTGTGGAAGACTTCAGGGAC |  |
| Os06g21570QRTR | TCCAGGTGCTAATATGCCGTTG |  |
| Os06g46940QRTF | TAATGAGCCTTACAACTTTGCGA |  |
| Os06g46940QRTR | CATTTGAGAACGGTTCATACCAT |  |
| Os07g46280QRTF | AACATCGTAGAGGCGTTTGCA |  |
| Os07g46280QRTR | GAAGCCATTGTCATATCCTAGAGC |  |
| Os08g39860QRTF | CAGTGGACTTTTACCATCGCTACAA |  |
| Os08g39860QRTR | CTCTTTGTTGATTCCTCCACTGATG |  |
| Os08g39870QRTF | TTCTCTCTGCTCTACTGTTCATCGC |  |
| Os08g39870QRTR | TCGACAGCACCCTCGTACTGATAC |  |
| Os09g31410QRTF | AAGGCAGATTGTTCCTGTTCTTGT |  |
| Os09g31410QRTR | GCGTCCATGTTCATGTCTTTGA |  |
| Os09g31430QRTF | TGAGTGGAGGAATAAACAAAGAAGG |  |
| Os09g31430QRTR | TGTCCCAGTGGAAGATAGTGACAAA |  |
| Os09g33680QRTF | ATTCCTCAGCAGTGTCCCTCTAC |  |
| Os09g33680QRTR | CCAACATTCTTCCTCATTACCG |  |
| Os09g33690QRTF | TGAATGAGCCGAACATAGAGCC |  |
| Os09g33690QRTR | ATACCACCAGCCCAGCAGAGT |  |
| Os09g33710QRTF | ATCGTCGTCGTGTTCCTCTTG |  |
| Os09g33710QRTR | TGATGAAGGTGTCCCAAATGC |  |
| Os10g17650QRTF | GAACCGCTTCATCGGCTTATC |  |
| Os10g17650QRTR | GTTGACCTCACCAGTGCCATTT |  |
| Os11g08120QRTF | CATCTTTGGCACAGGTTCAGCA |  |
| Os11g08120QRTR | CAAGGACCCGTTTGGCAGG |  |
| Os11g45710QRTF | GAGTTGAAACTTGCTAAGGAGACCG |  |
| Os11g45710QRTR | AAGGTGGAAGTGAGTGATGAAACAGA |  |
| Os12g23170QRTF | GGTATGAGCCACTCACCGACAA |  |
| Os12g23170QRTR | GGCAGCCTTTCCTTAACAGCAT |  |

RT, reverse-transcription PCR, qRT, real-time quantitative PCR.
