# Supplementary material for: A Phylogenetically Informed Comparison of GH1 Hydrolases between Arabidopsis and Rice Response to Stressors
Source: Front Plant Sci. 2017 Mar 24;8:350. doi: 10.3389/fpls.2017.00350 (PMC5364172; doi:10.3389/fpls.2017.00350)
Supplement: Supplementary file 2 [file Image1.PDF]

Figure S1: Alignment of rice and Arabidopsis GH1 proteins with large gap regions removed for phylogenetic analysis.

```
                      *        20         *        40         *        60         *
Os10bglu34 : LTRKSFPNGFVFGTASSAYQYEGAVKEDGRGPTIWDKFAH-TFGK-IIDFSNADVAVDQYHRFEEDIQLMADMGM :  73
Os3bglu6   : LTRGSFPEGFVFGTASAAYQYEGAVKEDGRGQTIWDTFAH-TFGK-ITDFSNADVAVDQYHRFEEDIQLMADMGM :  73
AtBGLU40   : ISRGSFPKGFVFGTASSAFQHEGAVKAEGRGPTIWDTFSH-TFGK-ITDFSNADVAVDQYHRYEEDVQLMKNMGM :  73
Os6bglu25  : ISRADFPPGFIFGTASSAYQYEGAVNEGQRGPTIWDTLTK-RPGR-VIDFSNADVAVDHYHRYKEDVELMNDIGM :  73
AtBGLU41   : ISRANFPDGFVFGTASSAYQFEGAVKEGNKGESIWDTFTKEKPGK-ILDFSNADTTVDQYHRFHNDIDLMKDLRM :  74
Os1bglu4   : T-RGDFPDGFVFGVATSAYQIEGARREGGKGDNIWDVFTE-NKER-ILDGSSGEVAVDHYHRYKEDIELMASLGF :  72
AtBGLU42   : THRSNFPSTFTFGVATSAYQIEGGWNEGKKGPSIWDKFTH-IEGK-ILDGSNGDVAVDHYHRYKEDVDLIGQLGF :  73
Os1bglu1   : LSRRSFPAGFVFGTAASAYQVEGMALKDGRGPSIWDAFVK-TPGE-IANNATADVTVDEYHRYKEDVNIMKSMGF :  73
Os7bglu26  : LSRRAFPEGFVFGTAASAYQVEGMAKQGGRGPSIWDAFIE-KPGT-IPNNATADVTVDEYHRYKEDVNIMKNMGF :  73
AtBGLU43   : LNRKSFPEGFLFGTATSAYQVEGETHQDGRGPSIWDAFVK-IPGK-IANNATAEITVDQYHRYKEDVDLMQNLNI :  73
AtBGLU44   : LSRQSFPKGFVFGTATSAYQVEGETHQDGRGPSIWDAFVK-IPGK-IAKNATAEITVDQYHRYKEDVDLMKKLNF :  73
Os3bglu8   : LSRAAFPKGFVFGTATSAFQVEGMAASGGRGPSIWDPFVH-TPGN-IAGNGNADVTTDEYHRYKEDVDLLKSLNF :  73
Os3bglu7   : LSRAAFPKRFVFGTATSAYQVEGMAASGGRGPSIWDAFAH-TPGN-VAGNQNGDVATDQYHRYKEDVNLMKSLNF :  73
Os12bglu38 : LTRETFPAGFVFGTASSAYQVEGNALQYGRGPCIWDTFLM-QPGV-TPDNSTANVTVDEYHRYMDDVDNMVRVGF :  73
Os11bglu37 : FSRYSFPKDFIFGTGSAAYQYEGAYKEGGKGPSIWDTFTH-VPGK-ILNNDTGDVASDFYHRYKEDVNLLKDMNM :  73
Os11bglu35 : FSRYSFPKDFIFGTGSAAYQYEGAYKEGGKGPSIWDTFTH-IPGK-ILNNDTGDVANDFHPDTREDVNLLKDMNM :  73
Os9bglu30  : FTRYSFPKDFIFGTGSAAYQYEGAYKEGGKGPSVWDNFTH-IPGK-ILNNDNGDVANDFYHRYKEDVSLLKDMNM :  73
Os9bglu29  : FNRYSFPKDFIFGTGSAAYQYEGAAKEGGRGPSVWDTFSH-IPGK-ILNGDTGDVADDFYHRYKEDVNLLKDMNM :  73
Os8bglu28  : LNRHSFPEGFLFGTGTSAYQYEGAVDK--RGQNIWDTFSR-IPGK-IADGSNADIANDFYHRYKEDLNLITAMNM :  71
Os8bglu27  : FNRFSFPEDFIFGTGSAAYQYEGAVNEGGRGPSIWDTYAH-IPGK-VEDGSNGDVAVDFYHRYKEDLNFVTDMNM :  73
Os4bglu10  : ISRRSFPKGFIFGTSSSSYQFEGAAAKGGRGPSIWDTFTHQYPDK-ITDKSNGDGACNSYHLYKEDVRIMKEMGM :  74
Os4bglu11  : ISRRSFPKGFIFGTSSSSYQFEGGAVLGGRGPSIWDTFTHQSPDK-ITDRSNGDVACDSYHLYKEDVRSMKEMGM :  74
Os4bglu9   : ISRRSFPKGFIFGTSSASYQCEGGAAEGGRGSSIWDTFTYQHPDK-IADKSNGDVADNTYHLYKEDVHMMKEMGM :  74
Os4bglu13  : ISRRSFPEGFIFGTASSSYQYEGGAREGGRGPSIWDTFTHQHPDK-IADKSNGDVAADSYHLYKEDVRIMKDMGV :  74
Os4bglu12  : VSRRSFPKGFIFGTASSSYQYEGGAAEGGRGPSIWDTFTHQHPEK-IADRSNGDVASDSYHLYKEDVRLMKDMGM :  74
Os6bglu24  : -RRSQFPEDFFFGTASSAYQYEGAVREGGRGPSIWDTFTHNHPEK-IANGSNGDIAIDSYHRYKEDVGIMKGLGL :  73
AtBGLU17   : LQRSSFPQDFRFGAASSAYQSEGAANVDGREPSIWDTFTKQYPEK-ISDGSNGDVADEFYYRFKEDVAHMKEIGL :  74
AtBGLU12   : LRRSDFPEDFIFGAATSAYQVEGAAHEDGRGPSIWDTFSEKYPEK-IKDGSNGSIASDSYHLYKEDVGLLHQIGF :  74
AtBGLU13   : LRRSDFPKDFIFGAATSAYQVEGAAHEDGRGPSIWDTFSEKYPEK-IKDGTNGSIASDSYHLYKEDVGLLHQIGF :  74
AtBGLU14   : LRKTDFPEDFIFGAATSAYQVEGAAQEDGRGPSIWDTFSEKYPEK-IKDGSNGSIADDSYHLYKEDVGLLHQIGF :  74
AtBGLU15   : LRRSDFPEDFIFGSATSAYQVEGGAHEDGRGPSIWDTFSEKYPEK-IKDGSNGSVADNSYHLYKEDVALLHQIGF :  74
AtBGLU16   : V------------------QVSG-------TPS-------------LK---NS----------QEDVNLLHQIGF :  24
AtBGLU18   : FSRLNFPEGFIWGTATAAFQVEGAVNEGCRGPSMWDTFTKKFP-H-RCENHNADVAVDFYHRYKEDIQLMKDLNT :  73
AtBGLU19   : LSRASFPEGFMFGTATAAFQVEGAVNEGCRGPSLWDIYTKKFP-H-RVKNHNADEAVDFYHRYKEDIQLMKKLNT :  73
AtBGLU20   : FTRANFPKGFIFGTATAAFQVEGAVNEGCRGPSMWDVYTKKFP-H-KCNYHNADVAVDFYHRYKEDIKLMKNLNT :  73
AtBGLU21   : LSRASFPNGFLFGTATAAFQVEGAINETCRGPALWDIYCRRNP-E-RCSGDHADVAVDFFHRYKEDIQLMKNLNT :  73
AtBGLU22   : LSRASFPNGFVFGTATAAFQVEGAINETCRGPALWDIFCKRNP-E-RCSGHNADVAVDFFHRYKEDIQLMKNLNT :  73
AtBGLU23   : LSRASFPEGFLFGTATAAYQVEGAINETCRGPALWDIYCRRYP-E-RCNNDNGDVAVDFFHRYKEDIQLMKNLNT :  73
AtBGLU24   : LSRAHFPKGFLFGTATAAYQVEGAVNETCRGPSVWDIYCKKYP-E-KCNGDNGTQAVDFFYRYKEDIQLMKNLNT :  73
AtBGLU25   : FGRGSFPDGFLFGATTSAFQHEGAAEEGGRGSSIWDSFTLKQHSE-SNNNLDGRLGVDFYHHYKEDVQLLKKLNM :  74
AtBGLU26   : KGRASFPKGFLFGTASSSYQYEGAVNEGARGQSVWDHFSNRFPHR-ISDSSDGNVAVDFYHRYKEDIKRMKDINM :  74
AtBGLU27   : FGRSDFPEGFLFGTASSAYQYEGARNEAPRGESVWDTFVRKYPER-NCYSN-ADQAIEFYNHYKDDIQRMKDINM :  73
AtBGLU28   : FDRHGFPDNFVFGTAASAFQYEGATSEGGKSPSIWDYFSHTFP-E-RTRMQNADVAVDFYHRYKDDIKLMKELNM :  73
AtBGLU29   : LDRSSFPDDFVFGTAISAFQSEGATSEGGKSPTIWDYFSHTFP-E-RTNMQNADVAVDFYHRYKDDIKLIEELNV :  73
AtBGLU30   : LDRHSFPDDFIFGTAASAFQYEGATSEGGKSPTIWDHFSLTYP-E-RTKMHNADVAIDFYHRYKDDIKLMKELNM :  73
AtBGLU31   : LNRYSFPPHFDFGVASSAYQYEGAVEEGGRSLSIWDNFTHAFP-E-RTNMDNGDVAVDFYHRYKEDIKLIKEMNM :  73
AtBGLU32   : LNRYSFPPHFDFGVASSAYQYEGAVEEGGRSPSIWDNFTHAFP-E-RTNMDNGDVAVDFYHRYKDDIKLIKEMNM :  73
AtBGLU33   : IHKQDFPADFIFGTSVSAYQVEGAKKGSGRGLTSWDEFTHMFPEK-VQQNGDGDEGVDFYTRYKDDIKLMKELNT :  74
AtBGLU34   : FNRTGFPRNFTFGAATSAYQIEGAAH---RALNGWDYFTHRYPEK-VPDRSSGDLACDSYDLYKDDVKLLKRMNV :  71
AtBGLU35   : FNRSGFPKNFTFGAATSAYQIEGAAH---RALNGWDYFTHRYPEK-VPDRSSADLACDSYDLYKDDVKLLKRMNV :  71
AtBGLU36   : ISQ----KNFTFGAATSAYQVEGAAH---RALNGWDYFTHRYPER-VSDRSIGDLACNSYDLYKDDVKLLKRMNV :  67
AtBGLU37   : FNKQDFESDFIFGVASSAYQIEGGRG---RGLNVWDGFTHRYPEKGGADLGNGDTTCDSYRTWQKDLDVMEELGV :  72
AtBGLU38   : FNSGNFEKGFIFGVASSAYQVEGGRG---RGLNVWDSFTHRFPEKGGADLGNGDTTCDSYTLWQKDIDVMDELNS :  72
AtBGLU39   : FNRKHFDDDFIF----------EGGKG---RGLNVWDGFTHRYPEKGGPDLGNGDSTCGSYEHWQKDIDVMTELGV :  63
Os4bglu18  : IHRSDFPASFLFGTATSSYQIEGAYLEGNKSLSNWDVFTHLPG--NIKDGSNGDIADDHYHRYEEDVELMNSLGV :  73
Os4bglu14  : VDRSQFPPDFLFGTSSSAYQVEGGYLEGNKGLSNWDVFTHKQG--TIEDGSNGDTANDHYHRYMEDIELMHSLGV :  73
Os4bglu16  : LRRDDFPPGFLFGAATSAYQIEGAYLDDNKGLNNWDVFTHTQAG-RISDGRNGDVADDHYHRYTEDVDILHNLGV :  74
AtBGLU45   : VDSSPFPSDFLFGTASSAYQYEGAFLTDGKSLNNWDVFTHKNPG-KILDKNNADRAVDQYNRFLEDIQLMSFLGV :  74
AtBGLU46   : -DSSPFPSDFLFGTASSAFQYEGAFLTDGKGLNNWDVFAHENPG-KIVDGSNGDIATDQYHRYMEDIQSMNFLGV :  73
AtBGLU47   : EETFHFPKNFLFGTASSAYQYEGAYLTDGKTLSNWDVFTN-ISG-KIADGSHGKVAVDHYHRYPGDLDLMEDLGV :  73
Os9bglu33  : LTRHDFPEGFVFGAGSSAFQVEGAAAEDGRKPSIWDTFINQG---YMPDGSNADVSADQYHHYKEDVKLMYDMGL :  72
Os9bglu32  : LTRHDFPEGFVFGAGTSAFQVEGAAAEDGRKPSIWDTFTHQG---YSPGGAIADVSADQYHLYKEDVKLMYDMGL :  72
Os9bglu31  : ITRADFPPEFIFGAGSSAYQVEGAFAEDGRKPSIWDTFSHSG---YSVDGATGDVTADQYHKYKEDVKLLQDMGV :  72
Os1bglu2   : YTRSDFPRDFVFGAATSAYQYDGAAAEDGRSPTIWDTFAHEGQ--KTKDKGTGDVAADGYHKYKGDVKLMAETGL :  73
Os1bglu3   : YTRNDFPADFVFGAATSAYQYEGAAAEDGRGASIWDTFTHAG---KMKDKSTGDVASDGYHKYKGDVKLMTETGL :  72
Os1bglu5   : YTRGDFPEDFVFGSATSSYQYEGGFDEDGRSPSNWDIFTHQG---KMPGRSTADVAADGYHKYKDDLKLMVDTNL :  72
Os5bglu22  : FTRQDFPGEFVFGAGTSAYQYEGATDEDGRSPSIWDTFTHAG---KMPDKSTGDMGAGGYHKYKEDVKLMSDTSL :  72
Os5bglu23  : FTRQDFPGDFVFGAGTSAYQYEGATGEDGRTPSIWDTFTHSG---RMADNSTGDRAAAGYHKYKEDVKLMSDTGL :  72
Os5bglu20  : FRRDDFPDGFAFGAGTAAYQYEGAAAEDGRTPSIWDTYTHSG---RHPEDGTGDVASDGYHKYKEDVKLMTEIGL :  72
Os5bglu21  : FTRDDFPHDFAFGAGTSAYQYEGGAAEDGRTPSIWDTYTHSG---RHPEDETGDVASDGYHKYKEDVKLMSEIGL :  72
Os5bglu19  : FTRDDFPDGFTFGAGTAAFQYEGAAAEDGRTPSIWDTYAHSW---RNPGGETGDVACDGYHKYKEDVMLMNETGL :  72
AtBGLU1    : YSRSDFPEGFVFGAGISAYQWEGAVDEDGRKPSVWDTFLHCR---K-MDN--GDIACDGYHKYKEDVQLMAETGL :  69
AtBGLU3    : -DKNDFPEGFIFGSATSAYQWEGAFDEDGRKPSVWDTFLHTR---N-LSN--GDITSDGYHKYKEDVKLMVETGL :  68
AtBGLU4    : FSRSDYPEGFVFGAGTSAYQWEGAAAEDGRKPSLWDTLCHSR---D-QGN--GDIACDGYHKYKDDVKLMVDTNL :  69
AtBGLU5    : FSRSDFPEGFLFGAGTSAYQWEGAAAEDGRKPSVWDTLCYSR---N-IGN--GDVTCDGYHKYKEDVKLMVDTNL :  69
AtBGLU6    : FSRCDFPEGFVFGSSTSAYQWEGAVAEDGRKPSVWDRFCHSH---NNQGN--GDITCDGYHKYKEDVKLMVDTNL :  70
AtBGLU7    : FTRNDFPNDFLFGAATSAYQWEGAFDEDGKSPSVWDTTSHCD---SGSNN--GDIACDGYHKYKEDVMLMAEMGL :  70
AtBGLU8    : FTRNDFPEDFLFGAGTSAYQWEGAANEDGRTPSVWDTTSHCY---NGSN---GDIACDGYHKYKEDVKLMAEMGL :  69
```

```
AtBGLU9      : FTRNSFPKDFLFGAATSAYQWEGAVAEDGRTPSVWDTFSNSY---DTG---NGDVTSDGYHKYKEDVKLMATMGL :  69
AtBGLU10     : FTRNNFPKDFLFGAATSAYQWEGAVAEDGRTPSVWDTFSHTY---NRGNLGNGDITSDGYHKYKEDVKLMAEMGL :  72
AtBGLU11     : YSRNDFPPGFVFGSGTSAYQVEGAADEDGRTPSIWDVFAHAG---H-SGVAAGNVACDQYHKYKEDVKLMADMGL :  71
Osbglu39     : -MNTSLPANFLWGNSVSSMQTEGAWNEGGKGMSVYDIREAG------ENISDWKVATDSYHRYREDFDLMQDLGM :  68
Os11bglu36   : ADFRAFPDNFFFGLATARDQRDGGSQQSWRGENIGDGFEILAESGESAGGDNCSHNVAAWHRFDAELKLAKETGI :  75
                    p  f  g   a q eG          d                         h    d

                 80         *        100         *        120         *        140         *
Os10bglu34   : DAYRFSIAWSRIFPNGTGEVNQAGIDHYNKLINALLAKGIEPYVTLYHWDLPQALEDKYTGWLDRQIINDYAVYA : 148
Os3bglu6     : DAYRFSIAWSRIYPNGVGQVNQAGIDHYNKLIDALLAKGIQPYVTLYHWDLPQALEDKYKGWLDRQIVDDFAAYA : 148
AtBGLU40     : DAYRFSISWTRIFPNGVGHINEAGIDHYNKLINALLAKGIEPYVTLYHWDLPQALHDRYLGWLNPQIINDFAAYA : 148
Os6bglu25    : DAYRFSISWSRIFPNGTGEPNEEGLSYYNSLIDALLDKGIEPYVTLFHWDLPQALEDRYGGWLNSEIIEDFVQYA : 148
AtBGLU41     : DAYRFSISWSRIFPNGTGEVNPDGVKYYNSLIDALLAKGIKPYVTLYHWDLPQALEDRYEGWLSREVVDDFEHYA : 149
Os1bglu4     : RAYRFSISWPRIFPDGLGNVNEQGVAFYNDLINFMIEKGIEPYATLYHWDLPHNLQQTVGGWLSDKIVEYFALYA : 147
AtBGLU42     : GAYRFSISWSRIFPDGLGEVNEEGIAFYNDLINTLLEKGIQPYVTLYHWDLPSHLQEAIGGWTNRKIVDYFGLYA : 148
Os1bglu1     : DAYRFSISWSRIFPTGTGKVNWKGVAYYNRLINYMLKIGITPYANLYHYDLPEALEVQYGGLLNRKIVEAFADYA : 148
Os7bglu26    : DAYRFSISWSRIFPNGTGMVNQEGVDYYNRLIDYMVKKGIKPYANLYHYDLPLALHEQYLGWLSPNIVEAFADYA : 148
AtBGLU43     : DAYRFSISWSRIFPEGSGKINSNGVAYYNRLIDYLIEKGITPYANLYHYDLPLALEQKYQGLLSKQ--GRFCGLR : 146
AtBGLU44     : DAYRFSISWSRIFPEGSGKVNWKGVAYYNRLIDYMVQKGITPYANLYHYDLPLALENKYKGLLGRQVVKDFADYA : 148
Os3bglu8     : DAYRFSISWSRIFPDGEGKVNTEGVAYYNNLIDYVIKQGLIPYVNLNHYDLPLALQKKYEGWLSPKIVGVFSDYA : 148
Os3bglu7     : DAYRFSISWSRIFPDGEGRVNQEGVDYYNNLINYLLQKGITPYVNLYHYDLPLALEKKYGGWLNAKMADLFTEYA : 148
Os12bglu38   : DAYRFSISWSRIFPSGLGKINKDGVDYYHRLIDYMLANNIIPYVVLYHYDLPQVLHDQYKGWLHPRIVRDFVRFA : 148
Os11bglu37   : DAFRFSIAWTRILPNGSLGINREGVAFYNSLINDVIAKGMIPFVTIFHWDTPQALESKYGGFLSENIVKDYVDFA : 148
Os11bglu35   : DAFRFSIAWTRILPNGSLGINREGVAFYNSLINDVIAKGMIPFVTIFHWDTPQALESKYGGFLSENIVKDYADFA : 148
Os9bglu30    : DAFRFSIAWTRILPNGSLGINKEGVAFYNSLINDVIAKGMIPFVTIFHWDTPLALESKYGGFLSEDIVKEYVDFA : 148
Os9bglu29    : DAFRFSISWSRILPNGTLGVNKEGVAFYNNLINEIIAKGMKPFVTIFHWDTPQALESKYGGFLSENIIKDYVDFA : 148
Os8bglu28    : DSFRFSIAWSRILPNGTIGINKEGVEFYNSLINEVIAKGLKPFVTIFHFDTPQALEDKYGGFLSENIVKDYVDYA : 146
Os8bglu27    : DAFRFSIAWSRILPNGTIGINKEGIAFYNSLINEVISRGLKPFVTIFHFDTPQALEDKYRSFLSENIVKDFVDYA : 148
Os4bglu10    : DAYRFSISWSRILPNGSLGVNREGINYYNNLINELLSKEVQPFATLFHFDTPQALEDKYKGFLSPNIINDYKDYA : 149
Os4bglu11    : DAYRFSISWSRILPS-ALGVNREGISYYNNLINELLSKGVQPFVTLFHWDSPQALEDKYKGFLSPNIINDYKEYA : 148
Os4bglu9     : DAYRFSISWSRILPNGSLGVNIEGINYYNNLINELLLKGVQSFVTLFHYDTPQALEDKYNGFLSPNIINDYKDYA : 149
Os4bglu13    : DAYRFSISWTRILPNGSLGINREGISYYNNLINELLLKGVQPFVTLFHWDSPQALEDKYNGFLSPNIINDYKEYA : 149
Os4bglu12    : DAYRFSISWTRILPNGSLGVNKEGIKYYNNLINELLSKGVQPFITLFHWDSPQALEDKYNGFLSPNIINDFKDYA : 149
Os6bglu24    : NAYRFSVSWPRILPNGKLGVNLEGIKYYNNLIDELISKGVEPFVTLFHWDSPQALEQQYGGFLSNLIVEDFRDYA : 148
AtBGLU17     : DSFRFSISWSRILPRGTVGVNQAGINFYNHLINELISNGIRPLVTLFHWDTPQALEDEYGGFLNPQIVKDFVEYV : 149
AtBGLU12     : DAYRFSISWSRILPRENLGINQAGIDYYNNLINELLSKGIKPFATIFHWDTPQSLEDAYGGFLGAEIVNDFRDYA : 149
AtBGLU13     : GAYRFSISWSRILPRGNLGINQAGIDYYNNLINELLSKGIKPFATIFHWDTPQSLEDAYGGFFGAEIVNDFRDYA : 149
AtBGLU14     : NAYRFSISWSRILPRGNLGINQAGIDYYNNLINELLSKGIKPFATIFHWDTPQDLEDAYGGFRGAEIVNDFRDYA : 149
AtBGLU15     : NAYRFSISWSRILPRGNLGINQAGIDYYNNLINELLSKGIKPFATMFHWDTPQALEDAYGGFRGAEIVNDFRDYA : 149
AtBGLU16     : DAYRFSISWSRILPRGTLGINQAGIEYYNNLINQLISKGVKPFVTLFHWDLPDALENAYGGLLGDEFVNDFRDYA :  99
AtBGLU18     : DAFRLSIAWPRIFPHGRMGINKVGVQFYHDLIDELLKNNIIPLVTVFHWDTPQDLEDEYGGFLSGRIVQDFTEYA : 148
AtBGLU19     : DGFRLSISWPRIFPHGRMGISKEGVQFYHDLIDELLKNDITPLVTVFHWDTPADLEDEYGGFLSERIVPDFVEYA : 148
AtBGLU20     : DGFRFSIAWPRIFPHGRMGISKAGVQYYHDLIDELLANGITPLVTVFHWDTPQDLEDEYGGFLSDRIIKDFTEYA : 148
AtBGLU21     : DAFRLSIAWSRIFPHGRKGVSQAGVQFYHELIDELLKN--VPFVTVFHWDTPQDLEDEYGGFLSQNIVKDFREYA : 146
AtBGLU22     : DAFRLSIAWSRIFPHGRKGVSQAGVKFYHDLIDELLKNGIIPFVTVFHWDTPQDLEDEYGGFLSENIVKDFREYA : 148
AtBGLU23     : DAFRMSIAWPRIFPHGRKGVSQAGVQFYHDLIDELIKNGITPFVTVFHWDTPQDLEDEYGGFLSERIVKDFREYA : 148
AtBGLU24     : DSFRLSISWTRIFPHGREGVSKSGVQFYHDLIDELKRNGIIPFVTVFHWDTPQTLENEYGGFLSAHIVKDFREYA : 148
AtBGLU25     : DAFRFSISWSRIFPHGKKGVSETGVKFYNDLINELIANGVTPLVTLFQWDVPQALEDEYGGFLSDRILEDFRDFA : 149
AtBGLU26     : DSFRLSIAWPRVLPYGKRGVSEEGIKFYNDVIDELLANEITPLVTIFHWDIPQDLEDEYGGFLSEQIIDDFRDYA : 149
AtBGLU27     : DAFRFSISWPRIFPLGKKGVNKEGIQFYNDLIDELLANGITPLATLFHWDTPQALEDEYSGFLSEEAVDDFKDFA : 148
AtBGLU28     : DAFRFSISWARLIPSGKVGVNKEGVEFYKALIDELVANGIEPSMTLYHWDHPQSLEDEYGGFLSPQIVEDFRDFS : 148
AtBGLU29     : DAFRFSISWARLIPSGKVGVNKEGVQFYKALIDELIANGIQPSVTLYHWDHPQALEDEYGGFLNPQIIEDFRNFA : 148
AtBGLU30     : DAFRFSISWSRLIPSGKLGVNKEGVQFYKDLIDELLANDIQPSMTLYHWDHPQSLEDEYGGFLSPKIVEDFRDFA : 148
AtBGLU31     : DSFRFSLSWSRILPSGKLGVNKEGVQFYKNLIDELIENGIKPFVTIYHWDIPQALDDEYGSFLSPRIIDDFRNYA : 148
AtBGLU32     : DSFRFSLSWSRILPSGKLGVNKEGVQFYKNLIDELIKNGIKPFVTIYHWDIPQALDDEYGSFLSPRIIDDFRNFA : 148
AtBGLU33     : NGFRFSISWTRILPYGTIGVNEEGVKFYNDLINELLANGIQPSVTLFHWESPLALEMEYGGFLNERIVEDFREFA : 149
AtBGLU34     : QAYRLSIAWSRVLPKGRLGVDENGITYYNNLINELKANGIEPYVTIFHWDVPQTLEDEYGGFLSTRIVEDYTNYA : 146
AtBGLU35     : QAYRLSIAWSRVLPKGRLGVDENGITYYNNLINELKANGIEPYVTIFHWDVPQTLEDEYGGFLSTRIVEDYTNYA : 146
AtBGLU36     : QAYRFSIAWSRVLPKGRLGVDENGITYYNNLINELKANGIEPFVTIFHWDVPQDFRRRIWRLLKP-TYSDFKNYA : 141
AtBGLU37     : KGYRFSFAWSRILPKGKRGINEDGINYYSGLIDGLIARNITPFVTLFHWDLPQSLQDEYEGFLDRTIIDDFKDYA : 147
AtBGLU38     : TGYRFSIAWSRLLPKGKRGVNPGAIKYYNGLIDGLVAKNMTPFVTLFHWDLPQTLQDEYNGFLNKTIVDDFKDYA : 147
AtBGLU39     : DGYRFSLAWSRIAPR--E--NQAGVKYYNDLIDGLLAKNITPFVTLFHWDLPQVLQDEYEGFLNHEIIDDFKDYA : 134
Os4bglu18    : NAYRFSISWSRILPKGRFGVNPAGIDFYNKLIDSILLKGIQPFVTLTHYDIPQELEDRYGAWLNAEIQSDFGHFA : 148
Os4bglu14    : NSYRFSISWARILPKGRFDVNPDGVAFYNALIDGLVQKGIQPFVTICHYDIPHELDERYGGWLSPEIQKDFSYFA : 148
Os4bglu16    : NSYRFSISWARILPRGRLGVNSAGIAFYNRLINALLQKGIQPFVTLNHFDIPHELETRYGGWLGAAIREEFEYYS : 149
AtBGLU45     : NSYRFSISWCRILPRGRFEINYLGIKYYNIFIDALISRGIKPFVTLNHVDYPQELEDRFQSWLNPEMQKEFGYLA : 149
AtBGLU46     : NSYRLSISWSRVLPNGRFVINYKGIKYYNNLIDALIKKGITPFVTLNHFDYPQELENRFKSWLSSEMQKDFGYLA : 148
AtBGLU47     : NSYRLSLSWARILPKGRFDVNMGGIDHYNRMINDILKTGIEPFVTLTHYDIPQELEYRYGSWLNPQIREDFEHYA : 148
Os9bglu33    : DAYRFSIAWPRLIPDGRGEINPKGLEYYNNLIDELIMHGIQPHVTIYHFDLPQALQDEYGGILSPRFIEDYSAYA : 147
Os9bglu32    : DAYRFSIAWPRLIPDGRGEINPKGLEYYNNLIDELIMHGIQPHVTIYHFDLPQALQDEYGGILSPRFIEDYTAYA : 147
Os9bglu31    : DAYRMSISWSRLIPDGRGAVNPKGLEYYNNLIDELLSHGIQPHVTIYHFDFPQALQDEYNGILSPRFVEDFTAYA : 147
Os1bglu2     : EAYKFSISWSRLIPNGRGAVNQEGLKYYNNVIDELAKRGIQPHIMLCHLDLPQALEDEYDGWLSPRIVDDFTAYA : 148
Os1bglu3     : EAYRFSISWSRLIPSGRGAVNQQGLKYYNNIIDELTKRGIQVHVMLYHLDLPQALEDEYAGWLSPRIVEDFTAYA : 147
Os1bglu5     : EAYRLSISWSRIIPNGRGDVNPKGLQYYNDIIDGLVKNGIQVHIMLYQLDLPQVLEDEYDGWLSPRILEDFKAYA : 147
Os5bglu22    : EAYRFSISWSRLIPRGRGPVNPKGLEYYNSLIDELVERGIEIHVTLYHLDFPQILEDEYHGWLSPRVIDDFTAYA : 147
Os5bglu23    : EAYRFSISWSRLIPRGRGPINPKGLEYYNDLIDKLVKRGIEIHVTLYHLDFPQALQDEYNGWLSPRIIEDFTAYA : 147
Os5bglu20    : EAYRFTISWSRLIPSGRGAVNPKGLQFYNNMINELVKAGIQIQVVLYHSDLPQSLQDEYGGWINPKIVDDFTAYA : 147
Os5bglu21    : EAYRFTISWSRLIPSGRGAVNLKALQFYNSMINELVKAGIQIHVVMYHMDLPQSLQDEYGGWISPKIVDDFTAYA : 147
Os5bglu19    : EAYRFTISWSRLIPSGRGAVNPKGLQFYNSMINELVKAGIQIHAVLYHIDLPQSLQDEYGGWVSPKVVDDFAAYA : 147
AtBGLU1      : HTFRFSISWSRLISNGRGSINPKGLQFYKNFIQELVKHGIEPHVTLHHYDFPQYLEDDYGGWTNRKIIKDFTAYA : 144
AtBGLU3      : DAFRFSISWSRLIPNGRGPVNPKGLQFYKNFIQELVSHGIEPHVTLFHYDHPQYLEDEYGGWINRRIIQDFTAYA : 143
AtBGLU4      : DAFRFSISWSRLIPNGRGPVNQKGLQFYKNLIQELVSHGIEPHVTLYHYDHPQSLEDEYGGWLNHRMIKDFTTYA : 144
```

```
AtBGLU5     : DAFRFSISWSRLIPNGRGSVNQKGLQFYKNLISELITHGIEPHVTLYHYDHPQYLEDEYGGWVNNMMIKDFTAYV : 144
AtBGLU6     : DAFRFSISWSRLIPNRRGPVNQKGLQFYKNLIQELVNHGIEPYVTLHHFDHPQYLEDEYEGWLNHMIVEDFTAYA : 145
AtBGLU7     : ESFRFSISWSRLIPNGRGRINPKGLLFYKNLIKELRSHGIEPQVTLYHYDLPQSLEDEYGGWINRKIIEDFTAFA : 145
AtBGLU8     : ESFRFSISWSRLIPNGRGRINPKGLLFYKNLIKELRSHGIEPHVTLYHYDLPQSLEDEYGGWINHKIIEDFTAFA : 144
AtBGLU9     : ESFRFSISWSRLIPNGRGLINPKGLLFYNNLIKDLKSHGIEPHVTLYHYDLPQSLEDEYGGWINRKIIEDFTAYA : 144
AtBGLU10    : ESFRFSISWSRLIPNGRGLINPKGLLFYKNLIKELISHGIEPHVTLYHYDLPQSLEDEYGGWINRKIIEDFTAYA : 147
AtBGLU11    : EAYRFSISWSRLLPSGRGPINPKGLQYYNNLIDELITHGIQPHVTLHHFDLPQALEDEYGGWLSQEIVRDFTAYA : 146
Osbglu39    : NCYRFQISWSRVCPQGDGAFNDEGIAFYDRFIDDLIARGIEPMICLYHFDMPLALAQEYNGFNDRRVMEAFIRYG : 143
Os11bglu36  : SVFRMGVDWARLMPEEELSVNFAALERYRWIIQRVREYGMKVMLTLFHHSLP-PWAGKYGGWKMEKTVTYFMDFV : 149
                54f   W R6 p g      g6  Y   I  6    g  p  t6 h d P  l    g       d5   a

                  160        *        180        *        200         *        220
Os10bglu34  : ETCFQAFGDRVKHWITFNEPHTVAVQAYDSG-MHAPGRCSVLLH------LYCKK--GNSGTEPYIVAHNMILSH : 214
Os3bglu6    : ETCFREFGDRVKHWITLNEPHTVAIQGYDAG-LQAPGRCSVLLH------LYCKA--GNSGTEPYVVAHHFILAH : 214
AtBGLU40    : EVCFQRFGDRVKHWITFNEPHTFAIQGYDVG-LQAPGRCTILFK------LTCRE--GNSSTEPYIVGHNVILTH : 214
Os6bglu25   : FTCFKEFGDRVKHWITFNEPYNFAIDGYDLG-IQAPGRCSILSH------VFCRE--GKSSTEPYIVAHNILLAH : 214
AtBGLU41    : FTCFKAFGDRVKYWITFNEPHGVSIQGYDTG-IQAPGRCSLLGH------WFCKK--GKSSVEPYIVAHNILLSH : 215
Os1bglu4    : EACFANFGDRVKHWITINEPLQTAVNGYGIG-HFAPGG------------------EGETARCYLAAHYQILAH : 203
AtBGLU42    : DACFANFGDRVKHWITLNEPLQTSVNGHCIG-IFAPGRN-----------------EKPLIEPYIVSHHQVLAH : 204
Os1bglu1    : EFCFKTFGDRVKNWMTFNEPRVVAALGYDDG-NFAPGRCT----------KCTAG---NSATEPYIVAHHLILSH : 209
Os7bglu26   : DFCFQTFGDRVKDWFTFNEPRCVAALGYDNG-FHAPGRCS----------GCDAG--GNSTTEPYLAAHHLILSH : 210
AtBGLU43    : RVLFQTFGDRVKNWMTFNEPRVVAALGYDNG-IFAPGRCSEAFG------NCTDG---NSATEPYIVAHHLILAH : 211
AtBGLU44    : EFCYKTFGDRVKNWMTFNEPRVVAALGYDNG-TKAPGRCSKAFG------NCTEG---NSATEPYIVTHHLILAH : 213
Os3bglu8    : EFCFKTYGDRVKNWFTFNEPRIVAALGHDTG-TDPPNRCT----------KCAAG--GNSATEPYIVAHNIILSH : 210
Os3bglu7    : DFCFKTFGNRVKHWFTFNEPRIVALLGYDQG-TNPPKRCT----------KCAAG--GNSATEPYIVAHNFLLSH : 210
Os12bglu38  : DFCFKTYGHKVKNWFTINEPRMMANHGYGDG-FFPPGRCT----------GCQPG--GNSATEPYIAAHNLLLSH : 210
Os11bglu37  : EVCFREFGDRVKYWTTFNEPFTYNAYGYGKG-VFAPGRCSPYVS-----KSCGAG---DSSREPYIVTHHIHLSH : 214
Os11bglu35  : EVCFHEFGDRVKYWTTFNEPFTYSAYGYGGG-VFASGRCAPYVS-----KSCGAG---DSSREPYIVTHHIHLSH : 214
Os9bglu30   : EVCFREFGDRVKYWFTFNEPFTYSAYGYGKG-VFAPGRCSSYVS-----KSCGVG---DSSREPYIVAHHIHLSH : 214
Os9bglu29   : EVCFREFGDRVKFWATFNEPWTYCSQGYGTG-IHALGRCSPYVS-----TSCAGG---DSSREPYIAAHHVILAH : 214
Os8bglu28   : DLCFSLFGDRVKLWNTFNEPTIFCMNGYATG-IMAPGRCSPYAS-----ASCAAGG--DSGREPYVAGHHLLVAH : 213
Os8bglu27   : DVCFREFGDRVKSWNTFNEPMIFCAGGYGSG-TKAPGRCSPYVS-----KKCAPG---DSGNEPYVAGHNLLLAH : 214
Os4bglu10   : EICFKEFGDRVKHWITFNEPWNFCSMGYASG-TMAPGRCSSWEK-----GKCRVG---DSGREPYTACHHQLLAH : 215
Os4bglu11   : ETCFKEFGDRVKHWITFNEPWTFCSMGYASG-IMAPGRCSSWEV-----GKCRVG---DSGREPYTACHHQLLAH : 214
Os4bglu9    : EICFKEFGDRVKHWITFNEPWIFCSKAYASG-TYAPGRCSPWEM-----GKCSVG---DSGREPYTACHHQLLAH : 215
Os4bglu13   : ETCFKEFGDRVKHWITFNEPLSFCVAGYASGGMFAPGRCSPWE------GNCSAG---DSGREPYTACHHQLLAH : 215
Os4bglu12   : EICFKEFGDRVKNWITFNEPWTFCSNGYATG-LFAPGRCSPWEK-----GNCSVG---DSGREPYTACHHQLLAH : 215
Os6bglu24   : DICFREFGDRVKYWITFNEPWSFSIGGYSNG-ILAPGRCSSQGK-----SGCSKG---DSGREPYIVAHNQLLAH : 214
AtBGLU17    : DICFKEFGDRVKEWITINEPNMFAVLGYNVG-NIAPGRCSSYV------QNCTVG---NSATEPYLVAHYLILSH : 214
AtBGLU12    : DICFKNFGDRVKHWMTLNEPLTVVQQGYVAG-VMAPGRCSKFTN-----PNCTAG---NGATEPYIVGHNLILAH : 215
AtBGLU13    : DICFKNFGDRVKHWMTLNEPLTVVQQGYVAG-VMAPGRCSKFTN-----PNCTAG---NGATEPYIVGHNLILAH : 215
AtBGLU14    : DICFKSFGDRVKHWITLNEPLTVVQQGYVAG-VMAPGRCSKFTN-----PNCTAG---NGATEPYIVGHNLILAH : 215
AtBGLU15    : DICFKNFGDRVKHWMTLNEPLTVVQQGYVAG-VMAPGRCSKFTN-----PNCTDG---NGATEPYIVGHNLILSH : 215
AtBGLU16    : ELCFQKFGDRVKQWTTLNEPYTMVHEGYITG-QKAPGRCSNFYK-----PDCLGG---DAATEPYIVGHNLLLAH : 165
AtBGLU18    : NFTFHEYGHKVKHWITFNEPWVFSRAGYDNG-KKAPGRCSPYIPGYG--QHCQD---GRSGYEAYQVSHNLLLSH : 217
AtBGLU19    : NFTFHEYGDKVKNWITFNEPWVFSRSGYDVG-KKAPGRCSPYVKEFG--KLCQD---GRSGFEPYVVSHNLLVGH : 217
AtBGLU20    : NFTFQEYGDKVKHWITFNEPWVFSRAGYDIG-NKAPGRCSKYIKEHG--EMCHD---GRSGHEAYIVSHNMLLAH : 217
AtBGLU21    : DYVFTEYGGKVKNWITFNEPWVFAHAGYDLG-KKAPGRCSRYVPG------CEDR-EGQSGKEAYLVSHNLLNAH : 213
AtBGLU22    : DYVFTEYGGKVKNWITFNEPWVFAHAGYDVG-KKAPGRCSRYLKG------CEDR-DGRSGYEAYLVSHNLLNAH : 215
AtBGLU23    : DFVFQEYGGKVKHWITFNEPWVFSHAGYDVG-KKAPGRCSSYVNAK-----CQD---GRSGYEAYLVTHNLLISH : 214
AtBGLU24    : EFVFKEYGGKVKHWITFNEPWVFAHAGYDVG-KKAPGRCSPYAKDETVKGDCLG---GRSGYEAYLVSHNLLNAH : 219
AtBGLU25    : QFAFNKYGDRVKHWVTINEPYEFSRGGYETG-EKAPGRCSKYVNEK-----CVA---GKSGHEVYTVSHNLLLAH : 215
AtBGLU26    : SLCFERFGDRVSLWCTMNEPWVYSVAGYDTG-RKAPGRCSKYVNG-----ASVA---GMSGYEAYIVSHNMLLAH : 215
AtBGLU27    : ALCFEEFGDRVKLWVTLNEPWVYSIGGYDTG-RKAPGRASKYMNE-----AAVA---GESGLEVYTVSHNLLLAH : 214
AtBGLU28    : RVCFEEFGDKVKMWTTINEPYVITVAGYDTG-NKAVGRCSKWVNS-----KCQG---GDSGTEPYIASHHLLLAH : 214
AtBGLU29    : RVCFENFGDKVKMWTTINEPYVISVAGYDTG-IKAVGRCSKWVNS-----RCQA---GDSAIEPYIVSHHLLLSH : 214
AtBGLU30    : RICFEEFGDKVKMWTTINEPYIMTVAGYDQG-NKAAGRCSKWVNE-----KCQA---GDSSTEPYIVSHHTLLAH : 214
AtBGLU31    : RFCFQEFGDKVSMWTTFNEPYVYSVSGYDAG-NKAMGRCSKWVNS-----LCIA---GDSGTEPYIVSHHLLLAH : 214
AtBGLU32    : RFCFQEFGDKVSMWTTLNEPYVYSVSGYDAG-NKAIGRCSKWVNS-----LCIA---GDSGTEPYIVSHHLLLAH : 214
AtBGLU33    : NFCFKEFGDRVKNWATFNEPSVYSVAGYSKG-KKAPGRCSKWQAP-----KCPT---GDSSEEPYIVAHNQILAH : 215
AtBGLU34    : ELLFQRFGDRVKFWITLNQPFSLATKGYGDG-SYPPGRCTG---------CELG---GDSGVEPYTVAHNQLLAH : 208
AtBGLU35    : ELLFQRFGDRVKFWITLNQPLSLALKGYGNG-SYPPGRCTG---------CELG---GDSGVEPYTVAHNQLLAH : 208
AtBGLU36    : ELLFQRFGDRVKFWITLNQPYSLAVKGYGDG-QYPPGRCTD---------CEFG---GDSGTEPYIVGHHELLAH : 203
AtBGLU37    : DLCFERFGDRVKHWITINQLFTVPTRGYALG-TDAPGRCSQWVDK-----RCYG---GDSSTEPYIVAHNQLLAH : 213
AtBGLU38    : DLCFELFGDRVKNWITINQLYTVPTRGYALG-TDAPGRCSPKIDV-----RCPG---GNSSTEPYIVAHNQLLAH : 213
AtBGLU39    : NLCFKIFGDRVKKWITINQLYTVPTRGYAMG-TDAP----------------------------EPYIVAHNQLLAH : 182
Os4bglu18   : DVCFGAFGDRVKYWTTFNEPNVAVRHGYMLG-TYPPSRCSPPFG------HCARGG--DSHAEPYVAAHNVILSH : 214
Os4bglu14   : EVCFKLFGDRIKFWTTFNQPNLSIKFSYMDG-FYSPGRCSEPFG------KCALG---NSSIEPYVAGHNIILSH : 213
Os4bglu16   : DVCFNAFGDRVRFWTTFNEPNLSTRHQYILG-EFPPNHCSPPFG------NCSSG---DSRREPYAAAHNILLSH : 214
AtBGLU45    : DICFKHFGNRVKYWTTLNEPNQQLILGYLTG-KFPPSRCSSPYG------NCSQG---NSETEPFIAAHNMILAH : 214
AtBGLU46    : DICFKHFGDRVKHWITINEPNQHISLAYRSG-LFPPARCSMPYG------NCTHG---NSETEPFIAAHNMILAH : 213
AtBGLU47    : NICFRHFGDRVKFWSTFNEPNVQVILGYRTG-TYPPSRCSKPFG------NCSCG---DSYIEPLVAAHNIILSH : 213
Os9bglu33   : EVCFKNFGDRVKHWATFNQPNIEPIGGFDAG-DRPPRRCSYPFG-----TNC---TGGDSSTEPYIVAHHLLLAH : 213
Os9bglu32   : EVCFKNFGDRVKHWVTVNEPNIEPIGGYDAG-VQPPRRCSYPFG-----TNC---TGGDSSTEPYIVAHHLLLAH : 213
Os9bglu31   : DVCFKNFGDRVKHWSTVNEPNIEPIGGYDQG-ILPPRRCSFPFGV----LSC---DNGNSTTEPYIVAHHLLLAH : 214
Os1bglu2    : DVCFREFGDRVLHWTTLAEPNIAALGGYDTG-VLSPGHCSDPFGL----TEC---TVGNSTVEPYIAAHNMILTH : 215
Os1bglu3    : DVCFREFGDRVSHWTILAEPNVAALGGYDTG-EFAPGRCSDPFGV----TKC---TVGNSSVEPYVAAHNMILTH : 214
Os1bglu5    : DVCFKEFGDRVAHWITIDEPNVASIGSYDSG-QLAPGRCSDPFGI----RKC---TVGNSSVEPYIAVHNMLLAH : 214
Os5bglu22   : DVCFREFGDRVRHWTTMDEPNVLSIAAYDSG-AFPPCRCSPPFGA----N-C---TAGNSTVEPYVVAHNSILAH : 213
Os5bglu23   : DVCFREFGDLVRHWTTVGEPNVLSIAGYDSG-VIPPCRCSPPFGT----S-C---AAGDSTVEPYVAAHNSILAH : 213
Os5bglu20   : DVCFREFGDRVAHWTTVLEPNVMAQGCYDTG-TLPPNHCSYPFG-----SNC---TGGNSTVEPYIFIHHNLLAH : 213
Os5bglu21   : DVCFREFGDRVVHWTTVLEPNAMAQAGYDMG-ILPPNRCSYPFG-----SNC---TAGNSSVEPYIFIHHSLLAH : 213
```

```
Os5bglu19  : DVCFREFGDRVAHWTTSIEPNVMAQSGYDDG-YLPPNRCSYPFGR----SNC---TLGNSTVEPYLFIHHTLLAH : 214
AtBGLU1    : DVCFREFGNHVKFWTTINEANIFTIGGYNDG-NSPPGRCSFPGR------NC---TLGNSSTETYIVGHNLLLAH : 209
AtBGLU3    : NVCFREFGHHVKFWTTINEANIFTIGGYNDG-ITPPGRCSSPGR------NC---SSGNSSTEPYIVGHNLLLAH : 208
AtBGLU4    : DVCFREFGNHVKLWTTINEANIFSIGGYNDG-DTPPGRCSKPSK------NC---SSGNSSIEPYIVGHNLLLAH : 209
AtBGLU5    : DVCFREFGNYVKFWTTVNEANVFTIGGYNDG-DTPPGRCSLPGK------NC---LLGNSSTETYIVGHNLLLAH : 209
AtBGLU6    : DVCFREFGNHVKFWTTINEGNIFSIGGYNDG-DSPPGRCSIPGQ------NC---LLGNSSTEPYIVGHNLLLAH : 210
AtBGLU7    : DVCFREFGEDVKLWTKINEATLFAIGSYGD--GMRYGHCP-P-------MNY---STANVCTETYIAGHNMLLAH : 207
AtBGLU8    : DVCFREFGEDVKLWTTINEATIFAFAFYGK--DVRYGNC----------------TTGNYCMETYIAGHNMLLAH : 201
AtBGLU9    : DVCFREFGEDVKLWTTINEATIFAIGSYDQG-TAPPGHCS-PNKF----VNC---STGNSSTEPYIAGHNILLAH : 210
AtBGLU10   : DVCFREFGEDVKLWTTINEATIFAIGSYDQG-ISPPGHCS-PNKF----INC---TSGNSSTEPYIAGHNILLAH : 213
AtBGLU11   : DTCFKEFGDRVSHWTTINEVNVFALGGYDQG-ITPPARCSPPFG-----LNC---TKGNSSIEPYIAVHNMLLAH : 212
Osbglu39   : KKMIDCFGDRVKYWLTFNEQNIFHMPEAFR----ISGYLKGE----------------QTLRELYELQHHAMVAH : 198
Os11bglu36 : RLVVDCVSNLVDYWVIFNEPHVFVMLTYCAG--AWPGGDP---N------AIEVATSTLPTGVYNQALHWMAIAH : 213
               c    g 6 W t 2p      g  g    pgrc            c          ep   H     H

                  *      240         *      260         *      280         *      300
Os10bglu34 : ATVSDIYRKKYKASQNGELGISFDVIWYEPMSNSTADIEAAKRAQEFQLGWFADPFFFGDY-PATMRSRVGS-RL : 287
Os3bglu6   : AAAASIYRTKYKATQNGQLGIAFDVMWFEPMSNTTIDIEAAKRAQEFQLGWFADPFFFGDY-PATMRARVGE-RL : 287
AtBGLU40   : ATVSDIYRKKYKAKQGGSLGIAFDVMWFEPESNKTEDIEAAQRAQDFQLGWFLDPLMFGDY-PSSMRSRVGS-RL : 287
Os6bglu25  : AGAFRAYEQHFKNEQGGLIGIALNSRWYEPFSNADEDTEAAARAMDFELGWFLDPLMFGHY-PPSMQKLAGD-RL : 287
AtBGLU41   : AAAYHTYQRNFKEKQRGQIGISLDAKWYEPMSDCDEDKDAARRAMDFGLGWFMDPLINGDY-PASMKSLVEE-RL : 288
Os1bglu4   : AAAVDVYRRKFKAVQGGEVGLVVDCEWAEPFSEKTEDQVAAERRLDFQLGWYLDPIYFGDY-PESMRQRLGD-DL : 276
AtBGLU42   : ATAVSIYRSKYKESQGGQIGLSVDCEWAEPNSEKPEDKVAADRRIDFQLGWFLDPLFFGDY-PASMRQKLGD-NL : 277
Os1bglu1   : ASAVQRYRHKYQHIQKGKIGILLDFVWYEGLTNSTADQAAAQRSRDFHVGWFLHPIIYGEY-PKSLQVIVKE-RL : 282
Os7bglu26  : AAAVKRYREKYQLYQKGRIGILLDFVWYEPFSDSNADRAAAQRARDFHLGWFLDPIIHGRY-PYSMLEIVKD-RM : 283
AtBGLU43   : AAAVQRYRQNYQEKQKGRVGILLDFVWFEPLTSSQADNDAAQRARDFHVGWFIHPIVYGEY-PNTLQNIVKE-RL : 284
AtBGLU44   : AAAVQRYRKYYQAKQKGRVGILLDFVWYEPLTRSKADNLAAQRARDFHIGWFIHPLVYGEY-PKTMQNIVKE-RL : 286
Os3bglu8   : ATAVDRYRNKFQASQKGKIGIVLDFNWYEPLTNSTEDQAAAQRARDFHVGWFLDPLINGQY-PKNMRDIVKE-RL : 283
Os3bglu7   : AAAVARYRTKYQAAQQGKVGIVLDFNWYEALSNSTEDQAAAQRARDFHIGWYLDPLINGHY-SQIMQDLVKD-RL : 283
Os12bglu38 : AAAVRTYRDKYQAIQKGKIGILLDFVWYEPLTDKEEDHAAAHRAREFTLGWYLHPITYGHY-PETMQNAVKE-RL : 283
Os11bglu37 : AAADLPY----QPTQKGQIGMVVMTHWFVPYDDTVAHRGAVQRSLDFMFGWFMDPLVHGDY-PGTMRGWLGD-RL : 283
Os11bglu35 : AAVVHLYRTRYQPTQKGQIGMVVVTHWFVPYDDTAADRGAVQRSLDFMFGWFMDPLVHGDY-PGTMRGWLGD-RL : 287
Os9bglu30  : AAAVQLYRTKYQPTQKGQIGMVVVTHWFVPYDNSDADRGAVQRSLDFIYGWFMDPIVHGDY-PGTMRGWLGN-RL : 287
Os9bglu29  : ATAVHLYRTKYQPTQHGQIGITAVSHWFVPYNDTAADRRVVQRSLDFMYGWFLDPIVHGDY-PGTMRGWLGA-RL : 287
Os8bglu28  : AEAVRLYRARYRAAHGGEVGITQVSHWFEPYDASAADRRAARRALDFMLGWFMHPVAHGEY-PPAMRRLVGG-RL : 286
Os8bglu27  : AEAVRLYRQKYQATQKGQIGITQVSHWFVPYSDAAADKHAVRRSLDFMYGWFMDPIVFGDY-PGTMRKLVGD-RL : 287
Os4bglu10  : AETVRLYKEKYQFLQKGRIGIILNSEWFVPFSQSKSSNDAARRVLDFMLGWFMDPLIRGDY-PLSMRELVGN-RL : 288
Os4bglu11  : AETVRLYKEKYQ-LQKGKIGIILNADWFVPLSQSKSSSDAARRALDFMLGWFMDPLIRGDY-PLSMRELVGN-RL : 286
Os4bglu9   : AETVRLYREKYQ-LQKGKIGIIVNSQWYVPFSQSKTNKDAARRVLDFVLGWLMDPLIRGDY-PLNMRELVGN-RL : 287
Os4bglu13  : AETVRLYKEKYQ-LQKGKIGITLVSNWFVPFSRSKSNIDAARRALDFMLGWFMDPLIRGEY-PLSMRELVRN-RL : 287
Os4bglu12  : AETVRLYKAKYQ-LQKGKIGITLVSHWFVPFSRSKSNNDAAKRAIDFMFGWFMDPLIRGDY-PLSMRGLVGN-RL : 287
Os6bglu24  : AAVVQIYREKYQ-GQKGKIGITIVSNWMIPYEDSKEDKHATKRALDFMYGWFMDPLTKGDY-PVSMRTLVGN-RL : 286
AtBGLU17   : AATVQLYREKYQSFHGGTIGMTIQTYWMIPKYNTPACREAAKRALDFFFGWFADPITYGDY-PKTMRELVGN-RL : 287
AtBGLU12   : GEAVKVYREKYKASQKGQVGIALNAGWNLPYSESAEDRLAAARAMAFTFDYFMEPLVTGKY-PIDMVNYVKGGRL : 289
AtBGLU13   : GEAVKVYREKYKASQKGQVGIALNAGWNLPYTESAEDRLAAARAMAFTFDYFMEPLVTGKY-PVDMVNNVKDGRL : 289
AtBGLU14   : GEAIKVYRKKYKASQKGQVGIALNAGWNLPYTESAEDRLAAARAMAFTFDYFMEPLVTGKY-PVDMVNNVKGGRL : 289
AtBGLU15   : GAAVQVYREKYKASQQGQVGIALNAGWNLPYTESPKDRLAAARAMAFTFDYFMEPLVTGKY-PVDMVNNVKG-RL : 288
AtBGLU16   : GVAVKVYREKYQATQKGEIGIALNTAWHYPYSDSYADRLAATRATAFTFDYFMEPIVYGRY-PIEMVSHVKDGRL : 239
AtBGLU18   : AYAVDAFRNCKQCA-GGKIGIAHSPAWFEPQDL-EHVGGSIERVLDFILGWHLAPTTYGDY-PQSMKDRVGH-RL : 288
AtBGLU19   : AEAVDAFRKCEKCK-GGKIGIAHSPAWFEPEDV-EGGQATVNRVLDFVIGWHLDPTTFGDY-PQSMKDAVGS-RL : 288
AtBGLU20   : ADAVDAFRKCDKCK-GGKIGIAHSPAWFEAHELDEEHETPVTGLIDFILGWHLHPTTYGDY-PQSMKDHIGH-RL : 289
AtBGLU21   : AEAVEVFR--QKVK-GGKIGIAHSPAWFEPHDLDSNDAPTVSRVLDFMLGWHLEPTTSGDY-PQIMKDLLGY-RL : 283
AtBGLU22   : AEAVEVFR--QKVK-GGKIGIAHSPAWFEPHDLDSNDVPTVSRVLDFMLGWHLDPTTFGDY-PQIMKDLLGH-RL : 285
AtBGLU23   : AEAVEAYRKCEKCK-GGKIGIAHSPAWFEAHDLDSQDGASIDRALDFILGWHLDTTTFGDY-PQIMKDIVGH-RL : 286
AtBGLU24   : AEAVEAFRQCEKCK-GGKIGIAHSPAWFEPHDFDEQSGATIDRALDFIMGWHLDTTMFGDY-PQTMKDIVGH-RL : 291
AtBGLU25   : AEAVEEFRKCGKCT-GGKIGIVQSPMWFEPYDKSSPSEEIVKRAMDFTLGWHMEPITHGDY-PQAMKDVVGS-RL : 287
AtBGLU26   : AEAVEVFRKCDHIK-NGQIGIAHNPLWYEPYDPDPDDVEGCNRAMDFMLGWHQHPTACGDY-PETMKKSVGD-RL : 287
AtBGLU27   : AEAVEVFRNNPKCK-DGKIGIAHCPVWFEPYDSCPKDIEACERAMEFMFGWHMDPTVYGDY-PAVMKKSIGK-RL : 286
AtBGLU28   : AAAVQEFRKCNKTQ-DGQIGIVLSPLWFEPYDSSPADNEAVKRALATELDWHLDPVIHGDY-PEMMKKLAGN-RL : 286
AtBGLU29   : AAAVQEFRNCNKTLQDGKIGIVISPWWLEPYDSSSADKEAVERGLPLELEWHLNPVIYGDY-PETMKKHVGN-RL : 287
AtBGLU30   : AAAVEEFRKCEKTSHDGQIGIVLSPRWFEPYHSSTDDKEAAERALAFEIGWHLDPVIHGDY-PEIVKKYAGN-KL : 287
AtBGLU31   : AAAVEEFRKCDKISQDSKIGIVLSPYWFEPYDSSNADKEAVERALAFNIGWHLSPLVFGDY-PETIKISAGN-RL : 287
AtBGLU32   : AAAVEEFRKCDKISQDAKIGIVLSPYWFEPYDISESDKEAVERALVFNIGWHLSPLVFGDY-PETIKTTAGN-RL : 287
AtBGLU33   : LAAVDEFRNCKKVEGGGKIGIVLVSHWFEPKDPSSEDVKAARRSLEYQLGWFLRPLTYGQY-PAEMLEDVNI-RL : 288
AtBGLU34   : AKTVSLYRKRYQKFQGGKIGTTLIGRWFAPLNESELDKAAAKRAFDFFVGWFLDPLVYGKY-PTIMREMVGD-RL : 281
AtBGLU35   : AKTVSLYRKRYQKFQGGKIGTTLIGRWFVPLNESELDKAAAKRAFDFFVGWFLDPLVYGKY-PTIMREMVGD-RL : 281
AtBGLU36   : MEAVSLYRKRYQKFQGGKIGTTLIGRWFIPLNENDLDKAAAKREFDFSVLGSTGVRTISK---D--NERLGD-RL : 272
AtBGLU37   : ATVVDLYRTRYK-YQGGKIGPVMITRWFLPYDDTLESKQATWRAKEFFLGWFMEPLTKGKY-PYIMRKLVGN-RL : 285
AtBGLU38   : AAAVDVYRTKYKDDQKGMIGPVMITRWFLPFDHSQESKDATERAKIFFHGWFMGPLTEGKY-PDIMREYVGD-RL : 286
AtBGLU39   : AKVVHLYRKKYKPKQRGQIGVVMITRWFVPYDSTQANIDATERNKEFFLGWFMEPLTKGKY-PDIMRKLVGR-RL : 255
Os4bglu18  : ATAIEIYKRKYQSKQRGMIGMVLYSTWYEPLRDVPEDRLATERALAFETPWFLDPLVYGDY-PPEMRQILGG-RL : 287
Os4bglu14  : ANAVSVYRNKYQGKQGGQIGIALSITWYEPFRNTTIDLLAVKRALSFGASWFLDPILLGDY-PTEMREVLGQ-SL : 286
Os4bglu16  : AAAVHNYKTNYQAKQGGSIGIVIAVKWYEPLTNSTEDVRAARRALAFEVDWFLDPIFFGDY-PREMREILSS-NL : 287
AtBGLU45   : AKAVNIYKTKYQKEQKGSIGIVVQTSWFEPISDSNADKEAAERAQSFYSNWILDPVIYGKY-PKEMVDILGP-AL : 287
AtBGLU46   : AKAIQIYRTKYQREQKGKIGIVVQTSWFEPISDSIADKNAAERAQSFYSNWILDPVVYGKY-PEEMVNLLGS-AL : 286
AtBGLU47   : LAAVNLYRTKFQEQQRGQIGIVMNTIWFEPISDSLADRLAADRAQAFYLTWFLDPVVFGRY-PREMREILGD-DL : 286
Os9bglu33  : ASAVSIYRQKYQAIQGGQIGITLMVRWHEPYTDKTADAAAAIRMNEFHIGWFLHPLVHGDY-PPVMRSRVGV-RL : 286
Os9bglu32  : ASAVSIYRRKYQAIQGGQIGITLLGWWYEPYTDAVADAAAAIRMNEFHIGWFMNPLVHGDY-PPVMRSRVGA-RL : 286
Os9bglu31  : SSAVSLYREKYQATQGGQIGITLLGWWYEPGTQDPEDVAAAARMNDFHIGWYMHPLVYGDY-PPVMRKNVGS-RL : 287
Os1bglu2   : AAVVRLYREKYQALQKGIVGINMFSLWSYPLTNSIADLQAAQRYKDFSYGWILHPLVFGDY-PQVMKKTIGS-RL : 288
Os1bglu3   : AAVVRLYREKYQTLQKGIVGINVLSLWSYPLTDSTADLQAAQRYKDFTYGWILHPLVFGDY-PQVMKKAIGS-RL : 287
Os1bglu5   : ASVTKLYREKYQVAGKGIIGISVYTFWAYPLTNSTVDLEATKRCQDFIVHWVLRPLVFGDY-PQVMKNIVGS-RL : 287
```

```
Os5bglu22  : ASVTRLYRDKYQATQEGFVGMNIYSFWNYPFSSSSADIAATQRALDFMVGWILDPLVYGDY-PEIMKKKAGS-RI : 286
Os5bglu23  : ASAVRLYWDKYQAKQKGVVGTNIYSFWPYPLSRSCADIDAVQRVLDFTIGWILDPLVYGDY-PEIMKKQAGS-RI : 286
Os5bglu20  : ASAVRLYREKYQVAQKGIVGINIYSLWFYPLTDSAEDIGATERAKQFMYGWILHPLVFGDY-PETIKKVVGS-RL : 286
Os5bglu21  : ASAVRLYREKYKVAQKGIIGINIYSMWFYPFTDSAEDIGATERAKKFIYGWILHPLVFGDY-PDTMKKAAGS-RL : 286
Os5bglu19  : ASAVRLYREKHQAAQKGVVGMNIYSMWFYPLTESTEDIAATERVKDFMYGWILHPLVFGDY-PETMKKAAGS-RL : 287
AtBGLU1    : ASVSRLYKQKYKDIQGGSVGFSLFAMNFTPSTNSKDDEIATKRANDFYLGWMLEPLIYGDY-PDVMKRTIGS-RL : 282
AtBGLU3    : ASASRLYKQKYKDMQGGSVGFSLFSLGFTPSTSSKDDDIAVQRAKDFYFGWMLEPFIFGDY-PDEMKRTVGS-RL : 281
AtBGLU4    : ASVSRRYKQKYKDKQGGSIGFSLFILGLIPTTSSKDDATATQRAQDFYVGWFLRPLLFGDY-PDTMKRTIGS-RL : 282
AtBGLU5    : ASASRLYKQKYKDKQGGSIGFGLYLMGLTPSTSSKDDAIATQRAKDFYFGW------------------------ : 260
AtBGLU6    : ASVSRLYKQNYKDKQGGSIGFSLLTIGFSPSTSSKDDAIATQRANDFFNGWMLGPLIYGDY-PDTMKRIVGS-RM : 283
AtBGLU7    : SSASNLYKLKYKTKQRGSVGLSIYAYGLSPYTDSKDDETATERAEAFLFGWMLKPLVVGDY-PDIMKRTLGS-RL : 280
AtBGLU8    : ASASNLYKLKYKSKQRGSIGLSIFALGLTPYTNSKDDEIATQRAKAFLYGWMLKPLVFGDY-PDEMKRTLGS-RL : 274
AtBGLU9    : ASASKLYKLKYKSKQKGSIGLSIFAFGLSPYTNSKDDEIATQRAKTFLYGWMLKPLVFGDY-PDEMKKTVGS-RL : 283
AtBGLU10   : ASASKLYKLKYKSTQKGSIGLSIFAFGLSPYTNSKDDEIATQRAKAFFYGWMLKPLVFGDY-PDEMKRTVGS-RL : 286
AtBGLU11   : ASATILYKQQYKYKQHGSVGISVYTYGAVPLTNSVKDKQATARVNDFYIGWILHPLVFGDY-PETMKTNVGS-RL : 285
Osbglu39   : MSLTEYLHQTKPG---QLMGGMLAHQLIYPATCKPRDIFCAQQYDEFLNQNLLGVFAGQGYSPAVMAVVEQEGFG : 270
Os11bglu36 : SEAYDYIHSKSKNERKPIVGVAHHVSFTRP-----------------YGLFDVAAVALAN-------SLT--LF : 261
              a                 g 6G         p          a  r       g     p   g y p

                         *       320         *        340         *        360          *
Os10bglu34 : PKFTEKEAAL-VNGSLDFMGINHYTTFYTKDDQSTVIE---KLLNNTLADTATISVPFRNGQ-PIGDRANSIWLY : 357
Os3bglu6   : PRFTADEAAV-VKGALDFVGINHYTTYYTRHNNTNIIG---TLLNNTLADTGTVSLPFKNGK-PIGDRANSIWLY : 357
AtBGLU40   : PVFTGSQSSL-VKGSLDFVGINHYTTYYARNNATNLIG---TLLHDAVSDSGTVTLPFK-GLSTIGDRASSIWLY : 357
Os6bglu25  : PQFSTHASKL-VSGSLDFVGINHYTTLYARNDRLRIRK---LVMDDASTDSAVIPTAYRHGK-KIGETAASSWLH : 357
AtBGLU41   : PKITPEMYKT-IKGAFDYVGINHYTTLYARNDRTRIRK---LILQDASSDSAVITSSFRGGV-AIGERAGSSWLH : 358
Os1bglu4   : PTFSEKDKEF-IRNKIDFVGINHYTSRFIAHHQDPE-D---IYFYRVQQVERIEKWNT--GE-KIGERAASEWLF : 343
AtBGLU42   : PRFTPEEKEFMLQNSWDFLGLNHYTSRLISHVSNKEAE---SNFYQAQELERIVELEN--GD-LIGERAASDWLY : 346
Os1bglu1   : PKFTADEVHM-VKGSIDYVGINQYTAYYVRDQQPNATT---LPS-YSSDWHAAPIYER-DGV-PIGPRANSDWLY : 350
Os7bglu26  : PTFSDEESRM-VKDSIDYVGINHYTSFYMKDPGPWNLT---PTS-YQDDWHVGFAYER-NGV-PIGAQANSYWLY : 351
AtBGLU43   : PKFTEEEVKM-VKGSIDFVGINQYTTYFMSDPKISTTP---KDLGYQQDWNVTFNFAK-NGT-PIGPRAHSEWLY : 353
AtBGLU44   : PKFTEKEVKM-VKGSIDFVGINQYTTYYMSEPHPTTKP---KDLGYQQDWNVEFGFAK-LGK-PIGPRAYSSWLY : 355
Os3bglu8   : PTFTPEQAKL-VKGSADYFGINQYTANYMADQPAPQQA---ATS-YSSDWHVSFIFQR-NGV-PIGQQANSNWLY : 351
Os3bglu7   : PKFTPEQARL-VKGSADYIGINQYTASYMKGQQLMQQT---PTS-YSADWQVTYVFAK-NGK-PIGPQANSNWLY : 351
Os12bglu38 : PNFTREQSEM-IKGSADYIAINHYTTYYVS--HHVNKT---SIS-YLNDWDVKISYER-NGV-PIGKQAYSNWLY : 349
Os11bglu37 : PKLTLAQSAM-VKGSYDFIGINYYTTYYAKSMPP-PNS---NELSYDVDSRANTTGFR-NGK-PISPQEFTPIFF : 351
Os11bglu35 : PKFTPAQSAM-VKGSYDFIGINYYTTYYAKSVPP-PNS---NELSYDVDSRANTTGFR-NGK-PIGPQ-FTPIFF : 354
Os9bglu30  : PEFTPEQSAM-VKGSYDFIGVNYYTTYYAKSIPP-PNS---NELSYDLDNRANTTGFR-NGK-PIGPQEFTPIFF : 355
Os9bglu29  : PAFTAEQAAA-VRGSYDFIGVNYYTTYYAKSVPL-PSS---NRLSYDTDIRANTTGFR-NGK-PIGPQEFTPIFF : 355
Os8bglu28  : PAFTAEQSEM-LRGSFDFIGLNYYTSNYAVAAPP-PNK---LHPSYLTDNWVNATGYR-NSI-PIGPPAYTPIFF : 354
Os8bglu27  : PKFTAEQSEL-VKGSYDFIGLNYYTTNYAKSVLRRPSK---LKPAYATDNWVNQTAYR-NGV-PIGPPAFTKIFF : 356
Os4bglu10  : PEFSKEQSEM-VKGAFDFIGLNYYAASYADNDPP-SYG---HNNSYNTDSHAKITGSR-NGI-PIGPQAASFWFY : 356
Os4bglu11  : PEFSKEQSGM-VKGAFDFIGLNYYTSSYADNDPP-SHG---HNNSYNTDAHAKITGSR-NGI-PIGPQAASFWFH : 354
Os4bglu9   : PKFTKEQSEM-VKGAFDFIGLNYYSSSYAENVLP-SYG---LKNSYNTDFHARITGSR-NGT-LIGPQAASSWLH : 355
Os4bglu13  : PQFTKEQSEL-IKGSFDFIGLNYYTSNYAGSLPP-SNG---LNNSYSTDARANLTAVR-NGI-PIGPQAASPWLY : 355
Os4bglu12  : PQFTKEQSKL-VKGAFDFIGLNYYTANYADNLPP-SNG---LNNSYTTDSRANLTGVR-NGI-PIGPQAASPWLY : 355
Os6bglu24  : PRFTKEQSKA-INGSFDFIGLNYYTARYIQGTKQ-DSN---SHKSYSTDSLTNERVER-NGT-DIGPKAGSSWLY : 354
AtBGLU17   : PKFTKKQSKM-VRGSFDFFGLNYYTSRYVEDVMF-YAN---TNLSYTTDSRVNQTTEK-NGV-PVGEPTSADWLF : 355
AtBGLU12   : PTFTAKQSKM-LKGSYDFIGRNYYSSSYAKDVPC--SS---ENVTLFSDPCASVTGER-EGV-PIGPKAASDWLL : 356
AtBGLU13   : PTFTAKQSKM-LKGSYDFIGINYYSSSYAKDVPC--SS---ENVTLFSDPCASVTGER-EGV-PIGPKAASDWLL : 356
AtBGLU14   : PTFTSKQSNM-LKGSYDFIGINYYSSSYAKDVPC--SS---ENVTMFSDPCASVTGER-DG------------- : 343
AtBGLU15   : PIFTAQQSKM-LKGSYDFIGINYYSSTYAKDVPC--ST---KDVTMFSDPCASVTGER-DGV-PIGPKAASDWLL : 355
AtBGLU16   : PTFTPEESEM-LKGSYDFIGVNYYSSLYAKDVPC--AT---ENITMTTDSCVSLVGER-NGV-PIGPAAGSDWLL : 306
AtBGLU18   : PKFTEAEKKL-LKGSTDYVGMNYYTSVFAKEIS--PDP---KSPSWTTDSLVDWDSKSVDGY-KIGSKPFNGKLD : 356
AtBGLU19   : PRFTKAQKAK-LKDSTDFVGINYYTSFFAKADQK-VDS---RNPTWATDALVEFEPKTVDGSIKIGSQPNTAKMA : 358
AtBGLU20   : PKFTEAQKEK-LKNSADFVGINYYTSVFALHDEE-PDP---SQPSWQSDSLVDWEPRYVDKFNAFANKPDVAKVE : 359
AtBGLU21   : PQFTAAQKAK-LKDSTDFVGLNYYTSTFSNYNEK-PDP---SKPSWKQDSLVSWEPKNVDHS-AIGSMPLTAALP : 352
AtBGLU22   : PKFTSSQKAK-LKDSTDFVGLNYYTSTFSNHNEK-PDP---STPSWKQDSLVAWEPKNVDHS-AIGSQPLTAALP : 354
AtBGLU23   : PKFTEQKAK-LKASTDFVGLNYYTSVFSNHLEK-PDP---SKPRWMQDSLITWESKNAQNY-AIGSKPLTAALN : 355
AtBGLU24   : PKFTTEQIAK-LKNSADFVGINYYTSTFSKHLEK-PNH---AEPKFKQDSLVEWKNKNVNNI-TIGSKPETGPLP : 360
AtBGLU25   : PSFTPEQKEK-LKGSYDFVGINYFTSTFVAHTDN-VNP---EKPSWEADSRLQLHSNNVDGF-KIGSQPATAKYP : 356
AtBGLU26   : PSFTPEQSKK-LIGSCDYVGINYYSSLFVKSIKH-VDP---TQPTWRTDQGVDWMKTNIDGK-QIAKQGGSEWSF : 356
AtBGLU27   : PSFTAAQSKK-LRGSFDFVGVNYYSAFYVKNIDE-VNH---DKPNWRSDARIEWRKENNAGQ-TLGVRGGSEWDF : 355
AtBGLU28   : PSFTPEQSKM-LKNSSDFIGINYYTARYVAHIPQ-ADP---ARPRFVTDHQLQWRVTNHSNH-QFGPGEDRGILQ : 355
AtBGLU29   : PAFTPEQSKM-LINSSDFIGVNYYSIHFTAHLPH-IDH---TRPRFRTDHHFEKKLINRSNH-ETGPGDDRGKIH : 356
AtBGLU30   : PSFTVEQSKM-LQNSSDFVGINYYTARFAAHLPH-IDP---EKPRFKTDHHVEWKLTNHSGH-IIGPGEERGFLF : 356
AtBGLU31   : PSFTKEQSMM-VKNSFDFIGVNYYTARFVAHDLN-VDI---SRPRFMTDQHLQYKLTNRTGDTISLESDGTKILW : 357
AtBGLU32   : PSFTKEQSMM-LQNSFDFIGINYYTARFVAHDLH-VDL---SRPRFTTDQHLQYKLTNRSGDHISSESDGTKILW : 357
AtBGLU33   : REFTPEESEK-LRKSLDFVGLNYYGAFFSTPLAK-VNS---SQLNYETDLRVNWTVITNNLS-LPDLQTTSMGIV : 357
AtBGLU34   : PEFTPEQSAL-VKGSLDFLGLNYYVTQYATDAPP--P----TQLNAITDARVTLGFY-RNGV-PIGVVAP--SFV : 345
AtBGLU35   : PEFTPEESAL-VKGSLDFLGLNYYVSQYATDAPP--P----TQPNAITDARVTLGFY-RNGS-PIGVVAS--SFV : 345
AtBGLU36   : PKFTPKQSAL-LKGSLDFLGLNYYVTRYATYRPPPMP----TQHSVLTDSGVTIGFE-RNGV-SIGVKA---SIN : 337
AtBGLU37   : PKFNSTEARL-LKGSYDFLGLNYYVTQYAHALDPSPP----EKLTAMTDSLANLTSLDANGQ-PPGPPFSK-GSY : 353
AtBGLU38   : PEFSETEAAL-VKGSYDFLGLNYYVTQYAQNNQTIVPS---DVHTALMDSRTTLTSKNATGH-APGPPFNA-ASY : 355
AtBGLU39   : PKFNKKEAKL-VKGSYDFLGLNYYQTQYVYAIPANPP----NRLTVLNDSLSAFSYENKDG--PIGPWFNA-DSY : 322
Os4bglu18  : PSFSPEDRRK-LRYKLDFIGVNHYTTLYARDCMFSDCP---QGQ--ETQHALAAVTGESNGL-PIGTPTAMPTFY : 355
Os4bglu14  : PKFTSKQKNRLQSTKLDFIGLNHYTTCYVKDCIFSPCE---IDP--VNADARVFSLYERDGV-PIGKATGAPFFH : 355
Os4bglu16  : PKFTPEEKKLLQNNKVDFIGINHYTAIYAKDCIYSPCT---LDT--YEGNALVYAIGRRNGK-IIGKPTALHGYF : 356
AtBGLU45   : PQFSSNEVKNLEKSRADFVGINHYTSYFIQDCLTSACN---TGHGAFKAEGYALKLDRKGNV-TIGELTDVNWQH : 358
AtBGLU46   : PKFSSNEMNSLMSYKSDFLGINHYTSYFIQDCLITACN---SGDGASKSEGLALKLDRKGNV-SIGELTDVNWQH : 357
AtBGLU47   : PEFTKDDLKS-SKNALDFIGINQYTSRYAKDCLHSVCE---PGKGGSRAEGFVYANALKDGL-RLG--------- : 347
Os9bglu33  : PSITASDSEK-IRGSFDFIGINHYYVIFVQSIDAN----EQKLRDYYIDAGVQ-------------GEDDTENIQ : 343
Os9bglu32  : PSITASDSEK-IRGSFDFIGINHYFVIFVQSSDANH---DQKLRDYYVDAGVQENG---------GGGFDKEHYQ : 348
```

```
Os9bglu31   : PSFTAEESKR-VLESYDFVGFNHYVAIFVRADLSKL---DQSLRDYMGDAAVKYDLPFLKSNN--PLGLTSDFMT : 356
Os1bglu2    : PSFSQVQTEL-IKGAIDFIGINHYYSAYVNYRPLVE-----GVRDYVADRSVSAR-----VRFN----YEPTEYP : 348
Os1bglu3    : PSFSKVQTEL-VKGTLDFIGVNHYFSLYVSDLPLAK-----GVRDFIADRSVSCRGLLQGVRF-------PTRSM : 349
Os1bglu5    : PSFTKAQSED-VKGSLDFIGMNHYYSLYVNDRPLGK-----GTRDFVADISIYYRG----SKT-------PTSIG : 345
Os5bglu22   : PSFTEEQSEL-IRGSADFIGINHYTSVYISDASNGETV---GPRDYSADMAATFRISRN----Q----FVPTRLP : 349
Os5bglu23   : PSFTKEQSEL-IRGSADFIGINHYKSLYVSDGSNREKA---GLRDYNADMAAHFRGTT----------YAPSKTL : 347
Os5bglu20   : PFFSNHESEL-VTNAFDFVGLNHYSSVYTSNNNNVVKA---PLQDLTADIATLFRATKNDTPT----PEFLPGNT : 353
Os5bglu21   : PIFSNHESEM-VTNSFDFIGLNHYSSVYTSNNNNVVKA---PLQDLTADVATLFRVTKNDTPT----PVFVPGTI : 353
Os5bglu19   : PLFSDYESEL-VTNAFDFIGLNHYTSNYVSDNSNAVKA---PLQDVTDDISSLFWASKNSTPTLLLLRQFLPGTS : 358
AtBGLU1     : PVFSKEESEQ-VKGSSDFIGVIHYLTALVTNIDINPSL--SGIPDFNSDMG----------------------- : 330
AtBGLU3     : PVFSKEESEQ-VKGSSDFIGIIHYLAASVTSIKIKPSI--SGNPDFYSDMGVSMTWTVLGNFS-------AFEYA : 346
AtBGLU4     : PVFSEKESEQ-VKGSCDFVGVIHYHAASVTNIKSKPSL--SGNPDFYSYMET--DFGKSLDF----------QYA : 342
AtBGLU5     : -------------------------------------------------------------------------- :   -
AtBGLU6     : PVFSEEESEQ-VKGSSDYIGINHYLAASITNSKLKPSI--SGNPDFYSDMNV--ILSFFANFS-------SSEYD : 346
AtBGLU7     : PVFSEEESKQ-VKGSSDFVGVVHYNTFYVTNRPAPSLV-TSINKLFFADIGAYLIAAGNASL---------FEFD : 344
AtBGLU8     : PVFSEEESEQ-VKGSSDFVGIIHYTTFYVTNQPAPYIFPSSTNKDFFTDMGAYIISTGNSSS---------FVFD : 339
AtBGLU9     : PVFSEEESEQ-VKGSSDFIGIIHYTTFYVTNHQPSASLFPSMGEGFFKDMGVYIIPTGNSSF---------LVWE : 348
AtBGLU10    : PVFSEEESEQ-LKGSSDFIGIIHYTTFYVTN-KPSPSIFPSMNEGFFKDMGVYMISAANSSF---------LLWE : 350
AtBGLU11    : PAFTEEESEQ-VKGAFDFVGVINYMALYVKDNSSSLKP---NLQDFNTDIAVEMTLVGNTSIE-------N-EYA : 348
Osbglu39    : DIYRDEDLALLARTKNDFMAFSYYASKTLDSDVIPEGT---PVNYYLLHGEKNNPYLKATEWN----------WQ : 332
Os11bglu36  : PYVDSI-CD---KL--DFIGINYYGQEVISGPGLKLVDN----DEYSESGRG----------------------- : 303
              p              d  g n                         d

                380         *        400         *        420         *        440         *
Os10bglu34  : IVPRSM-RILMNYVKDRYNKP-TVYITENGMDDGNSPF--ISLKNALKD-DKRTKYHNDYLT-NLADSIREDGCD : 426
Os3bglu6    : IVPRGM-RSLMNYVKERYNSP-PVYITENGMDDSNNPF--ISIKDALKD-SKRIKYHNDYLT-NLAASIKEDGCD : 426
AtBGLU40    : IVPRGM-RSLMNYIKHRYGNP-PVFITENGMDDPNSIL--ISRKDALKD-AKRIKYHHDYLS-SLQASIKEDGCN : 426
Os6bglu25   : IVPWGM-FKLMKHVKEKYGNP-PVVITENGMDDANHPF--SRLEDVLQD-DKRIQYHNDYMS-NLLDAIRKEGCN : 426
AtBGLU41    : IVPWGI-RKLAVYVKDIYGNP-PVFITENGMDEKNSPF--IDMEKALKD-DKRIGFHRDYLS-NLSAAIRNDECD : 427
Os1bglu4    : IVPWGL-RKLLNYAAKRYGNP-VIYVTENGMDEEDDQS--ATLDQVLND-TTRVGYFKGYLA-SVAQAIK-DGAD : 411
AtBGLU42    : AVPWGI-RKTLNYMSKKYNHP-PIFITENGMDDEDDGS--ASIHDMLDD-KRRVDYFKSYLA-NVSQAIE-DGVD : 414
Os1bglu1    : IVPWGI-YKAVTYVKEKYGNP-TMFLSENGMDDPGN----VTIAQGVHD-TTRVAYYRSYIT-KLKEAID-DGAN : 416
Os7bglu26   : IVPWGI-NKAVTYVKETYGNP-TMILSENGMDQPGN----VSITQGVHD-TVRIRYYRNYIT-ELKKAID-DGAK : 417
AtBGLU43    : NVPWGM-YKALMYIEERYGNP-TMILSENGMDDPGN----ITLTQGLND-TTRVKYYRDYLV-QLKKAVD-DGAN : 419
AtBGLU44    : NVPWGM-YKALMYMKERYGNP-TMILSENGMDDPGN----VTLAQGLHD-TTRIKYYKDYLT-NLKKARD-DGAN : 421
Os3bglu8    : IVPTGM-YGAVNYIKEKYNNP-TIIISENGMDQSGN----LTREEFLHD-TERIEFYKNYLT-ELKKAID-DGAN : 417
Os3bglu7    : IVPWGM-YGCVNYIKQKYGNP-TVVITENGMDQPAN----LSRDQYLRD-TTRVHFYRSYLT-QLKKAID-EGAN : 417
Os12bglu38  : VVPWGI-YKAVMHVKEKYKDP-IIIIGENGIDQPGNETLP----GALYD-FFRIQYFDQYLH-ELKRAIK-DGAR : 415
Os11bglu37  : NYPPGI-REVLLYTKRRYNNP-AIF-TENGIDEGNNSTVP----EALRD-GHRIEFHSKHL--FVNHAIR-NGVN : 415
Os11bglu35  : NYPPGI-REVLLYTKRRYNNP-AIYITENGIDEGNNSTVP----EALRD-GHRIEFHSKHLQ-FVNHAIR-NGVN : 420
Os9bglu30   : NYPPGI-RELLLYTKRRYNNP-TIYVTENGIDEGNNSTLP----EALKD-GHRIEFHSKHLQ-FVNHAIK-NGVN : 421
Os9bglu29   : NYPPGL-RELLLYTKRRYNNP-IIYVTENGIAEGNNKSLPI--TEALKD-GHRIEFHSKHLQ-FVNHAIK-NGVN : 423
Os8bglu28   : NYPPGL-RELLLYVKRRYNNP-TIYITENGTDEANNSTIPI--SEALKD-ETRIGFHYKHLQ-FVHKAIQ-EGVK : 422
Os8bglu27   : TYAPGL-RELLLYTKRKYNDP-DIYIAENGTDEANNSTIPI--AEALKD-DNRISFHYQHLR-FTQLAIK-EGVK : 424
Os4bglu10   : IYPEGI-RELLLHIKENYGNP-TIYITENGVDEINNKTMRL--KEALKD-DIRIEYYHKHLL-ALLSAMR-DGAN : 424
Os4bglu11   : IYPEGI-CEMLLYVKENYGNP-TIYITENGVDEVNNKTMPL--EEALKD-DTRIEYYHKHLL-ALLSAMR-DGAN : 422
Os4bglu9    : IYPQGL-RELLLYIKENYGNP-TIFITENGVDEVNNKTMPL--KEALND-NTRIEYYHKHLL-ALRNAMR-DGAN : 423
Os4bglu13   : IYPQGF-RELVLYVKENYGNP-TIYITENGVDEFNNKTLPL--QEALKD-DTRIDYYHKHLL-SLLSAIR-DGAN : 423
Os4bglu12   : VYPQGF-RELVLYVKENYGNP-TIYITENGVDEFNNKTLPL--QEALKD-DARIEYYHKHLL-SLLSAIR-DGAN : 423
Os6bglu24   : IYPKGI-EELLLYTKRTYNNP-TIYITENGVDEVNNENLSL--KEALID-TTRIEFYRQHLF-HVQRALR-QGVD : 422
AtBGLU17    : ICPEGF-QDVLLYIKSKFQNP-VILVTENGMPSENDKSLSV--NIALND-EAKIKYHQLHLT-ALLEAVS-QGAD : 423
AtBGLU12    : IYPKGI-RDLLLYAKYKFKDP-VMYITENGRDEASTGKID------LKD-SERIDYYAQHLK-MVQDAIS-IGAN : 420
AtBGLU13    : IYPKGI-RDLLLYAKYKFKDP-VMYITENGRDEASTGKID------LKD-SERIDYYAQHLK-MVQDAIS-IGAN : 420
AtBGLU14    : ----GI-RDLILYAKYKFKDP-VMYITENGRDEASTGKIL------LKD-GDRIDYYARHLK-MVQDAIL-IGAN : 403
AtBGLU15    : IYPKGI-RDLVLYAKYKFKDP-VMYITENGRDEFSTNKIF------LKD-GDRIDYYARHLE-MVQDAIS-VGAN : 419
AtBGLU16    : IYPKGI-RDLLLHAKFRYNDP-VLYITENGVDEANIGKIF------LND-DLRIDYYAHHLK-MVSDAIS-IGVN : 370
AtBGLU18    : VYSKGL-RYLLKYIKDNYGDP-EVIIAENGYGEDLGEKHN-DVNFGTQD-HNRKYYIQRHLL-SMHDAICKDKVN : 426
AtBGLU19    : VYAKGI-RKLMKYIKDRYNSP-EIIITENGYGEDLGDKDT-DLSVALND-HNRKYYLQRHLL-ALNEAICEDKVN : 428
AtBGLU20    : VYAKGL-RSLLKYIKDKYGNP-EIMITENGYGEDLGEQDT-SLVVALSD-QHRTYYIQKHLL-SLHEAICDDKVN : 429
AtBGLU21    : VYAKGF-RKLLKYIKDKYANP-EIMIMENGYGDKLGTTD--SVDVGTAD-HNRKYYLQRHLL-AMNEAICIDKVR : 421
AtBGLU22    : VYAKGF-RSLLKYIKDKYANP-EIMIMENGYGDKLKDKD--SVEVGTAD-YNRKYYLQRHLL-AMNEAICIDKVR : 423
AtBGLU23    : VYSRGF-RSLLKYIKDKYANP-EIMIMENGYGEELGASD--SVAVGTAD-HNRKYYLQRHLL-SMQEAVCIDKVN : 424
AtBGLU24    : VYSTGF-RKVLKYVKDKYANP-EIIIMENGYGENLKEND--SVENGTAD-YNRESYLKKHLW-SMHKAICEDKVN : 429
AtBGLU25    : VCADGL-RKVLKYIKENYNDP-EIIVTGNGYKETLEEKD--VLPDALSD-SNRKYYHMRHLM-ALHGAVCEDKVN : 425
AtBGLU26    : TYPTGL-RNILKYVKKTYGNP-PILITENGYGEVAEQSQSLYMYNPSID-TERLEYIEGHIH-AIHQAIHEDGVR : 427
AtBGLU27    : LYPQGI-RKFLNYAKNKYESP-KFMITENGHCDIDYEKK--PKLSNLMD-LQRTEYHKKHLQ-SIQQAIQEDGVV : 424
AtBGLU28    : SHPEGL-RKVLNYIKDKYNNP-IVYIKENGINDYDDGTK--SREEILND-TFRISYHEDHLQ-QLQKAIIEDGCD : 424
AtBGLU29    : SHPEGL-RRVLNYIKDKYNNP-IVYVKENGIDHYDDGTK--SRETILKD-TFRISYHQDHLK-QVHKAIM----- : 420
AtBGLU30    : SHPEGL-RKVLNYIKERYNNM-PVYIKENGINDNDDGTK--PREEIVKD-TFRIEYHKTHFE-ELHKAIVEDGCD : 425
AtBGLU31    : SYPEGL-RKILNYIKNKYNNP-TIYITENGFDDYENGTV--TREEILED-TKRIEYHQKHLQ-ELQKAITEDGCD : 426
AtBGLU32    : SYPEGL-RKLLNYIKNKYNNP-TIYITENGFDDYENGSV--TREEIIED-TKRIEYHQNHLQ-QLQKAITEDGCN : 426
AtBGLU33    : IYPAGL-KNILKHIKDEYMDP-EIYIMENGMDEIDYGTK--NITEATND-YGRKEFIKSHIL-IMGKSIRMDKVR : 426
AtBGLU34    : YYPPGF-RQILNYIKDNYKNP-LTYITENGVADLDLG-N-VTLATALAD-NGRIQNHCSHLS--CLKCAMKDGCN : 413
AtBGLU35    : YYPPGF-RQILNYIKDNYKNP-LTYITENGVADLDLG-N-VTLATALAD-NGRIQNHCSHLS--CLKCAMKDGCN : 413
AtBGLU36    : FDVKDL-RHLVDFFLFVELLLSTRIPSDSKSHQKQELL-MLIANALAD-NGRIQFQCSHLS--CLKCAIEDGCN : 407
AtBGLU37    : YHPRGM-LNVMEHFKTKYGDP-LIYVTENGFSTSGGP---IPFTEAFHD-YNRIDYLCSHLC-FLRKAIK----- : 416
AtBGLU38    : YYPKGI-YYVMDYFKTTYGDP-LIYVTENGFSTPGD----EDFEKATAD-YKRIDYLCSHLC-FLSKVIKEKNVN : 422
AtBGLU39    : YHPRGI-LNVLEHFKTKYGNP-LVYITENGESGCNVK---GYFAWCLGDNYELWPSRSFHVSPFYLLHRKDKGAF : 392
Os4bglu18   : VVPDGI-EKMVKYFMRRYNNL-PMFITENGYAQGGDSYT--DAEDWIDD-EDRIEYLEGYLT-KLAKVIR-DGAD : 423
Os4bglu14   : DVPRGM-EEAVTYYKQRYNNT-PTYITENGYSQASNSNM--TAKDFTND-TGRITYIQGYLI-SLASAIR-KGAD : 423
Os4bglu16   : VVPEAM-EKVVMYVNDRYRNT-TIYITENGYSQHSDT----SMEDLIND-VERVNYMHDYLK-YLSSAIR-KGAN : 422
AtBGLU45    : IDPTGF-HKMLNYLKDRYPNM-PMFITENGFGDLQKPET--TDKELLND-TKRIQYMSGYLE-ALQAAMR-DGAN : 426
```

```
AtBGLU46    : IDPNGF-RKMLNYLKNRYHNI-PMYITENGFGQLQKPET--TVEELLHD-TKRIQYLSGYLD-ALKAAMR-DGAN : 425
AtBGLU47    : -EPVGM-EEMLMYATERYKNI-TLYVTENGFG-----EN--NTGVLLND-YQRVKFMSNYLD-ALKRAMR-KGAD : 409
Os9bglu33   : CHSWSL-GKVLNHLKLEYGNP-PVMIHENGY-----SDSPDIFGKINYNDDFRSAFLQGYLE-ALYLSVR-NGSN : 409
Os9bglu32   : LHPWAL-GKMLHHLKLKYGNP-PVMIHENGD-----ADSPETPGKIDYDDDFRSDFLQSYLE-VLHLSIR-NGSN : 414
Os9bglu31   : STPWAL-KKMLNHLQEKYKNP-IVMIHENGA-----AGQPDPSGGNTYDDDFRSQYLQDYIE-ATLQSIR-NGSN : 422
Os1bglu2    : NDPKGL-QLALEYLRESYGDF-PFYIEENGK----------GSTNDSLDDPDRVDYIKGYIG-GVLDAIR-NGVD : 409
Os1bglu3    : GDPHGL-QLMLQHLKESYGDL-PIYVQENGKLDLLPVFSGKASSNDSLDDTDRVDYIKGYIE-GVLNATR-NGVN : 420
Os1bglu5    : PDPQGL-RLMVQYLQETYGNL-PIYILENGY----------GSSNDTVHDNDRVDYLKSYIG-SILTAIR-NGAN : 406
Os5bglu22   : RDPKGL-QCMLEYLRDTYQGI-PVYIQENGF-------GHFGKDDDSLNDTDRVDYLSSYMG-STLAAIR-NGAN : 413
Os5bglu23   : SDPKGL-QCMLEYLKDTYEGI-PVYVQENGF-------GQFDKED-SLNDTERVEYLSSYMG-GTLAAIR-NGAN : 410
Os5bglu20   : VDPQGL-ENALEYIRENYGNL-TIYIQENGS----------GAPDGTLDDVERINYLQKYIA-ATLKAIR-NGAN : 414
Os5bglu21   : VDPRGL-EHALKYIREKYGNL-PIYIQENGI------ASGSGSSSETLDDVERINYLAKYIA-ATLKAIS--GAN : 417
Os5bglu19   : LDPRGL-ELALEYLQEKYGNL-LFYIQENGS----------GS-NATLDDVGRIDCLTQYIA-ATLRSIR-NGAN : 418
AtBGLU1     : ---------ILEYIKQSYGNP-PVYILENGK-------TMNQDLELQQKDTPRIEYLDAYIG-AVLKAVR-NGSD : 386
AtBGLU3     : VAPWAM-ESVLEYIKQSYGNP-PIYILENGT-------PMKQDLQLQQKDTPRIEYLHAYIA-AVLKSIR-NGSD : 410
AtBGLU4     : NTPWAM-EVVLEYIKQSYGNP-PVYILESAKQWQQIGTPMKQDSQLKQKDIPRVEYLHAYIG-GVLKSIR-NGSD : 413
AtBGLU5     : --------------------------------------------------------------------------- :   -
AtBGLU6     : VAPWAI-EAVL---------------------------------------------------------------- : 356
AtBGLU7     : AVPWGL-EGILQHIKQSYNNP-PIYILENGK-------PMKHGSTLQ--DTPRAEFIQAYIG-AVHNAIT-NGSD : 406
AtBGLU8     : AVPWGL-EGVLQHIKHRYNNP-PIYILENGS-------PMKHDSMLQ--DTPRVEYIQAYIG-AVLNAIK-SGSD : 401
AtBGLU9     : ATPWGL-EGILEYIKQSYNNP-PVYILENGM-------PMVRDSTLQ--DTQRIEYIQAYID-AVLNAMK-NGSD : 410
AtBGLU10    : ATPWGL-EGILEYIKQSYNNP-PIYILENGM-------PMGRDSTLQ--DTQRIEFIQAYIG-AMLNAIK-NGSD : 412
AtBGLU11    : NTPWSL-QQILLYVKETYGNP-PVYILENGQ-------MTPHSSSLV--DTTRVKYLSSYIK-AVLHSLSRKGSD : 411
Osbglu39    : IDPMGF-RTIITRYANDWRM--PVFPIENGI----GVIESWDGVNPIE-DTYRIDYHRAHID-AMKAAMFEDGAE : 398
Os11bglu36  : VYPDGIFRILIQFNERYKRLNIPFVITENGV----------SDET----DLIRKPYILEHLLATYAAII-MGVRV : 363
               p g                    e g                                        g

                     460        *       480        *       500
Os10bglu34 : VRGYFAWSLLDNWEWAAGY-TSRFGLYYVDYK--NRKRYPKNSVQWFKNLLAS : 476
Os3bglu6    : VRGYFAWSLLDNWEWAAGY-SSRFGLYFVDYKD-NLKRYPKNSVQWFKALLKT : 477
AtBGLU40    : VKGYFVWSLLDNWEWAAGY-SSRFGLYFVDYRD-NLKRYPKDSVHWFTSFLNS : 477
Os6bglu25   : VHGYFVWSLLDNWEWNSGY-TVRFGIYYIDYKN-NLTRIPKASVQWFSQVLAQ : 477
AtBGLU41    : VRGYFVWSLLDNWEWNSGY-TVRFGIYYVDYKN-NLTRIPKASARWFQTILSG : 478
Os1bglu4    : VRGYFAWSFLDNFEWAMGY-TKRFGIVYVDYKN-GLSRHPKASARWFSRFLKG : 462
AtBGLU42    : IKGYFAWSLLDNFEWAQGY-TKRFGLVYVDYKN-GLTRHPKSSAYWFMKFLKG : 465
Os1bglu1    : CIGYFAWSLLDNFEWKLGY-TSRFGLVYVDFR--TLRRYPKMSAYWFRDLVSS : 466
Os7bglu26   : VIGYFAWSLLDNFEWRLGY-TSRFGIVYVDYK--TLKRYPKDSAFWFKNMLSS : 467
AtBGLU43    : LTGYFAWSLLDNFEWLSGY-TSRFGIVYVDYK--DLKRYPKMSALWFKQLLKR : 469
AtBGLU44    : VVGYFAWSLLDNFEWLSGY-TSRFGIVYVDYK--TLKRYPKMSAQWFKQLLKR : 471
Os3bglu8    : VVAYFAWSLLDNFEWLSGY-TSKFGIVYVDFT--TLKRYPKDSANWFKNMLQA : 467
Os3bglu7    : VAGYFAWSLLDNFEWLSGY-TSKFGIVYVDFN--TLERHPKASAYWFRDMLKH : 467
Os12bglu38 : VTGYFAWSLLDNFEWRLGF-TSKFGIVYVDRS--TFTRYPKDSTRWFRKMIKS : 465
Os11bglu37 : VKGYFTWTFMDCFEWGDGY-LDLFGLIYVDRKT--LTRYRKDSSYWIEDFLRR : 465
Os11bglu35 : VKGYFT-TFMDCFEWGDGY-LDRFGLIYVDRKT--LTRYRKDSSYWIEDFLK- : 468
Os9bglu30   : VKGYFTWTFMDCFEWGDGY-LDRFGLIYVDRKT--LKRYRKESSYWIEDFLKR : 471
Os9bglu29   : VKGYFTWTFMDCFEWGDGY-LDRFGLIYIDRLN-NLKRYHKQSSYWIANFLKR : 474
Os8bglu28   : VKGYFTWTFMDCFEFGDGF-KDRFGLIYVDRAT--LARFRKKSSYWFADFLRR : 472
Os8bglu27   : VKGYFTWTFMDDFEWGDGY-TGRFGLIYVDRET--LKRYRKKSSYWFADFLKR : 474
Os4bglu10   : VKGYFAWSLLDNFEWSEGY-TVRFGINFVDYDN-GMKRYPKNSARWFKKFLRK : 475
Os4bglu11   : VKGYFAWSLLDNFEWAEGY-TVRFGINFVDYDD-GMKRYPKNSARWFKKFLQK : 473
Os4bglu9    : VKGYFAWSLLDNFEWADGY-TLRFGLNFVDYDD-GMKRHPKNSAHWFKKFLRE : 474
Os4bglu13   : VKGYFAWSLLDNFEWSNGY-TVRFGINFVDYND-GAKRYPKMSAHWFKEFLQK : 474
Os4bglu12   : VKGYFAWSLLDNFEWSNGY-TVRFGINFVDYND-GRKRYPKNSAHWFKKFLLK : 474
Os6bglu24   : VRGYFAWSLFDNFEWMDGY-SVRFGINYIDYKD-GLKRYPKRSSQWLQNFLHN : 473
AtBGLU17    : VRGYYIWSLMDDFEWEFGY-KYRYGLVYVDFQD-GLKRHLKSSALWYHHFLSN : 474
AtBGLU12    : VKGFFAWSLLDNFEWATGY-AVRFGLVYVDFNG-GRKRYPKKSAKWFKKLLNE : 471
AtBGLU13    : VKGFFAWSLLDNFEWATGY-SVRFGLVYVDFND-GRKRYPKKSAKWFRKLLSE : 471
AtBGLU14    : VKGFFAWSLLDNFEWASGY-TVRFGLVYVDFND-RRKRYLKKSAHWFRHLLNG : 454
AtBGLU15    : VKGFFAWSLLDNFEWAMGY-TVRFGLVYVDFKD-GCKRYPKKSAEWFRKLLNE : 470
AtBGLU16    : VKGYFAWSLMDNFEWSEGY-TVRFGLVFVDFED-GRKRYLKKSAKWFRRLLKG : 421
AtBGLU18    : VTGYFVWSLMDNFEWQDGY-KARFGLYYIDFQN-NLTRHQKVSGKWYSEFLKP : 477
AtBGLU19    : VTSYFLWSLMDNFEWQDGY-TARFGVYYIDFKN-NLTRMEKESAKWLSEFLKP : 479
AtBGLU20    : VTGYFHWSLMDNFEWQDGY-KARFGLYYVDFKN-NLTRHEKLSAQWYSSFLHD : 480
AtBGLU21    : VTGYFVWSLLDNFEWQDGY-KNRFGLYYVDFKN-NLTRYEKESAKYYKDFLAQ : 472
AtBGLU22    : VTGYFVWSLLDNFEWQDGY-NNRFGLYYVDFKN-NLTRYEKESAKYYKDFLGQ : 474
AtBGLU23    : VTGYFVWSLLDNFEWQDGY-KNRFGLYYVDFKN-NLTRYEKESGKYYKDFLSQ : 475
AtBGLU24    : VTGYFVWSLMDNFEWQDGF-KNRFGLYYIDYKN-NLTRHEKVSGKYYREFLSE : 480
AtBGLU25    : VKGYFVSSLMDGLEWEDGY-KTRSGLYYVDYGH-NMGRHEKQSAKWLSKLLEK : 476
AtBGLU26    : VEGYYVWSLLDNFEWNSGY-GVRYGLYYIDYKD-GLRRYPKMSALWLKEFLRF : 478
AtBGLU27    : VEGYFAWSLLDNCEWNAGY-GVRYGLFYVDYNN-GLKRFPKMSAMWFKEFLKR : 475
AtBGLU28    : VRGYYVWSLLDNFEWEHGY-STRFGVYYVDYDN-DLTRIPKDSVNWFKQFLDV : 475
AtBGLU29    : ----------------------------------------------------- :   -
AtBGLU30    : VRGYYAWSLMDNFEWEHGY-TARFGLYYVDFVN-GLKRYPKDSVKWFKRFLKK : 476
AtBGLU31    : VKGYFTWSLLDNFEWEHGY-AVRFGLYYVDYKN-GLQRHAKHSAMWFKHFLER : 477
AtBGLU32    : VKGYFTWSLLDNFEWEHGY-AVRFGLYYVDYKN-GLSRHAKNSAKWFKHFLQR : 477
AtBGLU33    : LKGYYIWSLMDNFEWDKGY-KVRFGLYYVDYND-NMKRYIRSSGKWLSEFLDS : 477
AtBGLU34    : VAGYFAWSLMDNYEFGNGY-TLRFGMNWVNFTN-PADRKEKASGKWFSKFLAK : 464
AtBGLU35    : VAGYFAWSLMDNYEFGNGY-TLRFGMNWVNFTN-PADRKEKASGKWFSKFLAK : 464
AtBGLU36    : VAGYFAWSLMDNYEFGNGY-TLRFDMNWVNFTN-PADRREKASGKWFSRFLAK : 458
AtBGLU37    : ----------------------------------------------------- :   -
AtBGLU38    : VKGYFAWSLGDNYEFCNGF-TVRFGLSYVDFANITGDRDLKASGKWFQKFINV : 474
AtBGLU39    : PS-FEA----------------------------------------------- : 397
```

```
Os4bglu18  : VRGYFAWSVVDNFEWLFGY-TLRFGLYYIDYRT--QERSPKLSALWYKEFLQN : 473
Os4bglu14  : VRGYFVWSLLDDFEWNFGY-TLRFGLYHVHYKT--LKRTPKLSVDWYRKFLTG : 473
Os4bglu16  : VGGYFAWSIVDNFEWVYGY-TVKFGLYQVDFDT--QERIPRMSAKWYRDFLTS : 472
AtBGLU45   : VKGYFVWSLLDNFEWLFGY-KVRFGLFHVDLTT--LKRSPKQSASWYKNYIEE : 476
AtBGLU46   : VKGYFAWSLLDNFEWLYGY-KVRFGLFHVDFTT--LKRTPKQSATWYKNFIEQ : 475
AtBGLU47   : VRGYFAWSLLDNFEWISGY-TIRFGMYHVDFST--QERTPRLSASWYKNFIFQ : 459
Os9bglu33  : TRGYFVWSMFDMFEFLYGY-RLRFGLCGVDFTAAARTRYLKNSARWYSGFLRG : 461
Os9bglu32  : TRGYFVWSLLDGFEFLSGY-GNRFGLCGVDFTAPARTRYVRSSARWYSDFLNG : 466
Os9bglu31  : VQGYFVWSFLDVFEYLFGY-RLRFGLYGVDFASPERTRYQRHSARWYAGFLRG : 474
Os1bglu2   : VRGYFVWSFVDVFELLEGY-QSRFGLYRVDFDDGAR-RRAAAASEALRSLVLG : 460
Os1bglu3   : ARGYFAWFFVDMFELLSGY-QTRYGLYRVDFDDAALPRRAKRSARWYRDFLKS : 472
Os1bglu5   : VKGYFVWSFVDVFEYLTGY-GQSYGLYRVDFADESRPRQARLSARWYSGFLKN : 458
Os5bglu22  : VKGYFVWSFLDVFELLAGY-HSPFGLHYVDFEDPNLPRQPKLSAHWYSKFLRG : 465
Os5bglu23  : VKGYFVWSFLDVFELFAGY-HSPFGLHHVDFEDPSLPRQPKLSAQWYSKFLRS : 462
Os5bglu20  : VKGYSMWSFIDIYEIFGGYNSWHYGLVAVDFGSTERRRQPRRSASWYSDFLKN : 467
Os5bglu21  : VKGYSMWSFVDLYELFGGYSTWHFGLVAVDFDSEKRRRQPRRSASWYSEFLKN : 470
Os5bglu19  : VKGYCVWSFMDQYEMFGDY-KAHFGIVAVDFGSEELTRQPRRSARWYSDFLKN : 470
AtBGLU1    : TRGYFVWSFMDLYELLNGY-KSSFGLYSVNFSDPHRKRSPKLSAHWYSGFLKG : 438
AtBGLU3    : TRGYFIWSFMDLYELVKGY-EFSFGLYSVNFSDPHRTRSPKLSAHWYSAFLKG : 462
AtBGLU4    : TRGYFVWSFMDLYELLGGY-EVGFGLYTVNFSDPHRKRSPKLSAYWYSDFLKG : 465
AtBGLU5    : ----------------------------------------------------- :   -
AtBGLU6    : ----------------------------------------------------- :   -
AtBGLU7    : TRGYFVWSMIDLYELIGRY-MTSYGMYYVNFSDPGRKRSPKLSASWYTGFLNG : 458
AtBGLU8    : TRGYFVWSLIDLFEVQVGY-KSSFGMYYVNFSDPGRKRSPKLSASWYTGFLNG : 453
AtBGLU9    : TRGYFVWSMVDVYEILSGY-TTSFGMYHVNFSDPGRKRTPKLSASWYTGFLNG : 462
AtBGLU10   : TRGYFVWSMIDLYELLSGY-TTSFGMYYVNFSDPGRKRTPKLSASWYTGFLNG : 464
AtBGLU11   : VKGYFQWSLMDVFELFGGY-ERSFGLLYVDFKDPSLKRSPKLSAHWYSSFLKG : 463
Osbglu39   : VIGYLGWGLIDILSSQGDM-RKRYGVVYVNRENHDLKRVPKKSYAWLKQVIHT : 450
Os11bglu36 : LGYLF-WTTSDNWEWADGY-GPKFGLVAVDRANN-LARKPRPSYFLFSRVVTT : 413
               g   w   d  e   g      g            r    s
```
