# Supplementary figures and images for: A Phylogenetically Informed Comparison of GH1 Hydrolases between Arabidopsis and Rice Response to Stressors
Source: Front Plant Sci. 2017 Mar 24;8:350. doi: 10.3389/fpls.2017.00350 (PMC5364172; doi:10.3389/fpls.2017.00350)

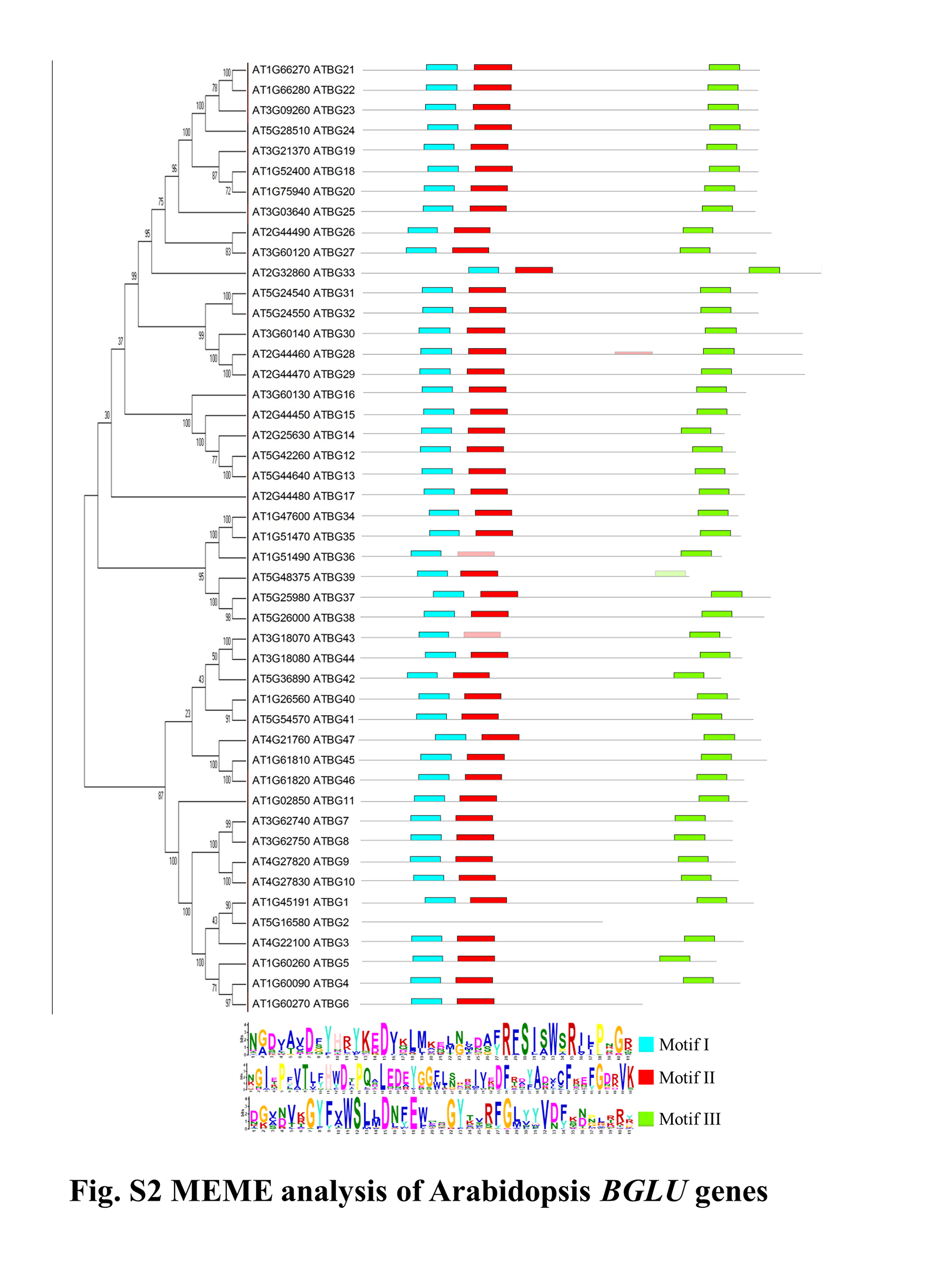

Supplement: Supplementary file 3 [file Image2.TIF]

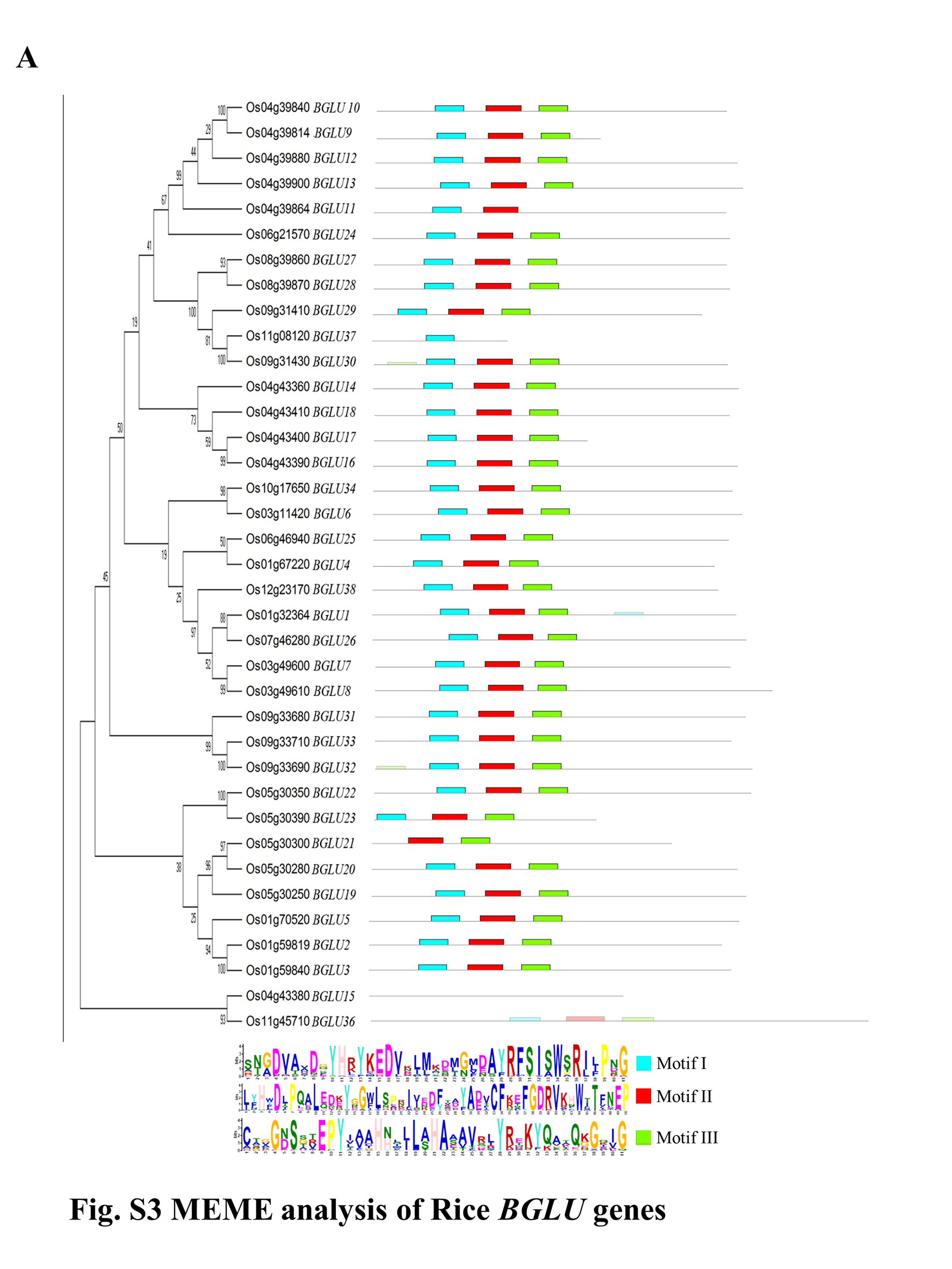

Supplement: Supplementary file 4 [file Image3.TIF]
